# How Prospecting for Informed Dispersal Shapes Biodiversity Patterns in a Metacommunity

**DOI:** 10.1002/ece3.73912

**Published:** 2026-07-07

**Authors:** Marie‐Sophie Rohwäder, Florian Jeltsch

**Affiliations:** ^1^ Plant Ecology and Nature Conservation University of Potsdam Potsdam Germany; ^2^ Berlin‐Brandenburg Institute of Advanced Biodiversity Research (BBIB) Berlin Germany

**Keywords:** competitive metacommunity, dispersal‐diversity‐relationship, individual‐based modelling, informed dispersal, prospecting

## Abstract

Prospecting for future breeding sites is a key aspect of making informed dispersal decisions. The aim of our study is to investigate the role of prospecting in mediating species co‐occurrence or competitive exclusion in a metacommunity, thereby altering the dispersal‐diversity relationship. An individual‐based model (IBM) was developed to simulate a small mammal metacommunity, composed of 10 theoretical species, incorporating local resource competition and regional breeding dispersal. The model included three scenarios of prospecting effort (measured as the number of patches prospected) across the metacommunity: uniform prospecting effort, interspecific differences in prospecting effort, and intraspecific differences in prospecting effort. We also tested the role of potential inheritance for the latter trait. The study further considered costs associated with prospecting and dispersal, including mortality risk during prospecting and integration costs in the new patch. Informed settlement significantly influenced the relationship between dispersal rate and metacommunity diversity. Low prospecting effort led to a steady increase in diversity with higher dispersal rates, while higher prospecting effort resulted in a hump‐shaped relationship. High prospecting effort led to rapid colonization of and settlement in high‐quality patches, increasing competition and reducing establishment success, especially for species with high resource demands. Introducing dispersal costs, particularly integration costs, reduced metacommunity diversity, but increased prospecting effort partially mitigated this loss. Species‐specific prospecting effort, correlated with body mass, resulted in a hump‐shaped relationship between dispersal rate and diversity, highlighting the role of prospecting for the competition‐colonization trade‐off. In contrast, when individual variation in prospecting behaviour was considered a heritable trait, the highest species diversity was observed at increasing dispersal rates, with prospecting efforts stabilising at low levels. The study demonstrates that informed dispersal, specifically prospecting effort, plays a crucial role in shaping metacommunity diversity by mediating competitive species interactions in an individual‐based metacommunity simulation model.

## Graphical abstract

This study uses an individual‐based model of a small mammal metacommunity to explore how prospecting effort influences competitive exclusion and the resulting dispersal‐diversity relationship. The results show that high prospecting effort can create a hump‐shaped relationship between dispersal and diversity by intensifying competition, a pattern that is significantly mediated by dispersal costs and the species‐specific or individual nature of prospecting behaviour. The work concludes that informed dispersal, through prospecting effort, is a crucial factor in shaping metacommunity diversity by mediating competitive interactions.

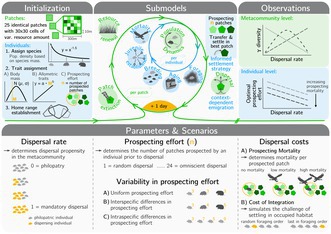

## 1 Introduction

Animals gather information and use it to reduce uncertainty and optimise their decision‐making (Dall et al. 2005; Schmidt et al. 2010). This is particularly evident in crucial decisions such as dispersal, which enables individuals to identify breeding sites that vary in space and time or to escape unfavourable conditions (Ronce 2007). Effectively navigating this process and choosing an optimal dispersal strategy requires several informed decisions that allow animals to move from poor to good conditions. Information thus plays an important role in the dispersal process, determining an individual's survival and reproductive success (Schjørring 2002).

The concept of ‘informed dispersal’, first proposed by Reed et al. (1999), captures the idea that individuals gather and integrate information at all stages of dispersal: emigration, transfer and settlement Clobert et al. (2009). A crucial component of informed dispersal is therefore the prospection phase, also known as exploratory movements or pre‐dispersal excursions (Ponchon 2024; Ponchon et al. 2013). Prospecting is defined as visits to sites other than one's current location to assess habitat quality. This behaviour is well‐studied in birds but also common across a wide range of taxa, including mammals, fish and invertebrates (Ponchon 2024). Prospecting allows individuals to gather information at both local and distant scales, with the goal of informing emigration and/or settlement decisions (Ponchon et al. 2013; Reed et al. 1999). The acquired information can support more adaptive decisions by optimising the trade‐off between the costs and benefits of dispersal (Bocedi et al. 2012). On the other hand, obtaining the information itself can be costly. Prospecting animals need to spend time and energy exploring potential future breeding sites. This entails the risk of mortality during the search period and can further cause deferred costs, which are incurred during prospecting but whose negative consequences are only experienced later (Stamps et al. 2005). Those risk for prospectors could for example entail an increase in energetic costs (Kingma et al. 2016) or a lower reproductive success caused by a delayed breeding start (Barba et al. 1995). By balancing the benefits and costs of making an informed decision, prospecting itself is coming under strong selection, often resulting in a lower prospecting effort and hence a semi‐informed dispersal strategy (Bocedi et al. 2012; Delgado et al. 2014; Ponchon et al. 2021).

While it was recognized that modelling the dispersal process needs to incorporate greater realism (Travis et al. 2012), many theoretical models of dispersal do not explicitly include the acquisition and use of information in dispersal decision‐making. This results in a dichotomy between either random or ‘omniscient’ dispersal (Bocedi et al. 2012; Delgado et al. 2014). Yet, as highlighted by Schmidt et al. (2010), scenarios of partially informed dispersal behave significantly differently from information‐free or perfect information scenarios. To date, only a few modelling studies have explicitly included prospecting as a mechanism of acquiring and using information in the dispersal process (Bocedi et al. 2012; Delgado et al. 2014; Ponchon et al. 2015, 2021; Enfjäll and Leimar 2009). Such efforts have demonstrated that prospecting has significant population consequences, reducing emigration rates (Enfjäll and Leimar 2009), enhancing population persistence (Ponchon et al. 2015) and impacting range expansion (Ponchon and Travis 2022).

However, there is a paucity of studies testing implications of prospecting for higher levels of organisation such as metacommunities. Yet, dispersal is not only important for individual fitness and population persistence, but is also a key determinant of biodiversity patterns (Jeltsch et al. 2013; Leibold et al. 2004; Schlägel et al. 2020). A long‐standing focus of metacommunity ecology has been the relationship between dispersal rate and diversity (Grainger and Gilbert 2016). Among other mechanisms, this relationship is shaped by the competition‐colonisation trade‐off, driving species coexistence under patch dynamics (Leibold et al. 2004). Prospecting for the optimal patch to settle will greatly affect an individual's colonisation success and thus contribute to this trade‐off. Species will clearly differ in their ability to acquire information, especially from more distant environments (Enfjäll and Leimar 2009). In addition to these interspecific differences, prospecting effort can also vary considerably between individuals of the same species (Fielding et al. 2023). As with other dispersal traits, this may be influenced by animal personality (Cote et al. 2010; Korsten et al. 2013; Nilsson et al. 2014), which is discussed to induce individual differences in prospecting effort (Kurvers et al. 2010; Martins et al. 2012). For example, bold individuals have been shown to be more exploratory than shy individuals, forming a common behavioural syndrome (Majelantle et al. 2022). Such intraspecific trait variability can strongly modulate species co‐occurrence and has recently been proposed as an important addition to the more generalised assumptions of modern coexistence theory (Jeltsch et al. 2019, 2025). With regard to individual differences in vertebrate behaviour, it is often of great interest and theoretical importance to establish whether these differences are heritable or occur randomly (Dochtermann et al. 2019). In a metacommunity context, individual organisms are sufficient to repopulate habitat patches and thus influence meta‐community dynamics as a whole. Therefore, the possible inheritance of prospecting efforts as a trait can be assumed to be highly relevant.

Overall, differences in prospecting effort either between or within species will clearly modulate the success to colonise and settle in the best patch and compete for spatially and temporally variable resources. Prospecting could thus have important implications for species co‐occurrence in a metacommunity context.

In this modelling study, we aim to determine how informed dispersal strategies, specifically prospecting effort and its possible inheritance, will shape the relationship between dispersal rate and diversity in a competitive metacommunity. We focus on settlement informed by prospecting, while the decision to emigrate depends solely on information about local density. The assumption of a one‐time, density‐dependent emigration is realistic for a number of species (Bowler and Benton 2005; Clobert et al. 2009) and can be found in numerous model studies (e.g., Bocedi et al. 2014; Hovestadt and Poethke 2006; Ponchon et al. 2021). Unfortunately, there is a lack of empirical studies on the search behaviour and decision‐making processes of small mammal dispersers when choosing where to settle. However, a radio‐telemetry study tracking dispersing juvenile brush mice ( *Peromyscus boylii* ) revealed that their search behaviour most often aligned with comparative decision rules, including the ‘best‐of‐n’ strategy (i.e., sampling multiple sites once before selecting the highest‐quality one) (Mabry and Stamps 2008). The more complex behaviour of repeatedly exploring the environment and potential new habitat patches before making a final emigration decision (i.e., emigration informed by prospecting), which can also be observed in some taxa (Clobert et al. 2009; Doran et al. 2013; Mabry and Stamps 2008; McMahon and Matter 2006) and would be the subject of a further study.

Using an individual‐based model of a small mammal community, we developed three scenarios for the distribution of prospecting effort, measured as the number of prospected patches, across the metacommunity:

**Table:** 

(A) Uniform prospecting effort	(B) Interspecific differences in prospecting effort	(C) Intraspecific differences in prospecting effort

For Scenario A, we would generally anticipate increased prospecting efforts to have a positive effect on metacommunity diversity, as prospecting enhances an animal's ability to locate and successfully colonise suitable habitat patches. However, for species that differ in their prospecting effort (Scenario B), the relationship between dispersal and diversity is hypothesised to become hump‐shaped. This should occur if less competitive species, with a stronger propensity for prospecting, become more successful colonizers compared to highly competitive but less discerning dispersers. In this case, prospecting facilitates a spatial mechanism for species' co‐occurrence arising from a competition‐colonization trade‐off. In addition, we also considered the costs associated with both prospecting and dispersal. While we would anticipate that prospecting could offset high dispersal costs, it also has its own costs. By understanding these key dynamics, we can better predict how landscape changes and habitat fragmentation may influence prospecting behaviour (Schmidt et al. 2010) and consequently metacommunity dynamics. Especially in the case of individual variability in prospecting effort (Scenario C), we would expect the greatest resilience to environmental change due to the elevated potential for adaptation to current conditions, especially if there is a heritable component to this trait, as there is for dispersal (Dochtermann et al. 2019; Saastamoinen et al. 2018). In our model, inheritance encompasses the transmission of prospecting efforts as a heritable trait from mothers to their offspring, with a certain degree of variability. This dynamic trait‐selection approach enables different levels of prospecting effort to emerge in various scenarios. The model does not explicitly consider genetic inheritance or potential evolutionary stable strategies.

## 2 The Model

**FIGURE 1:**
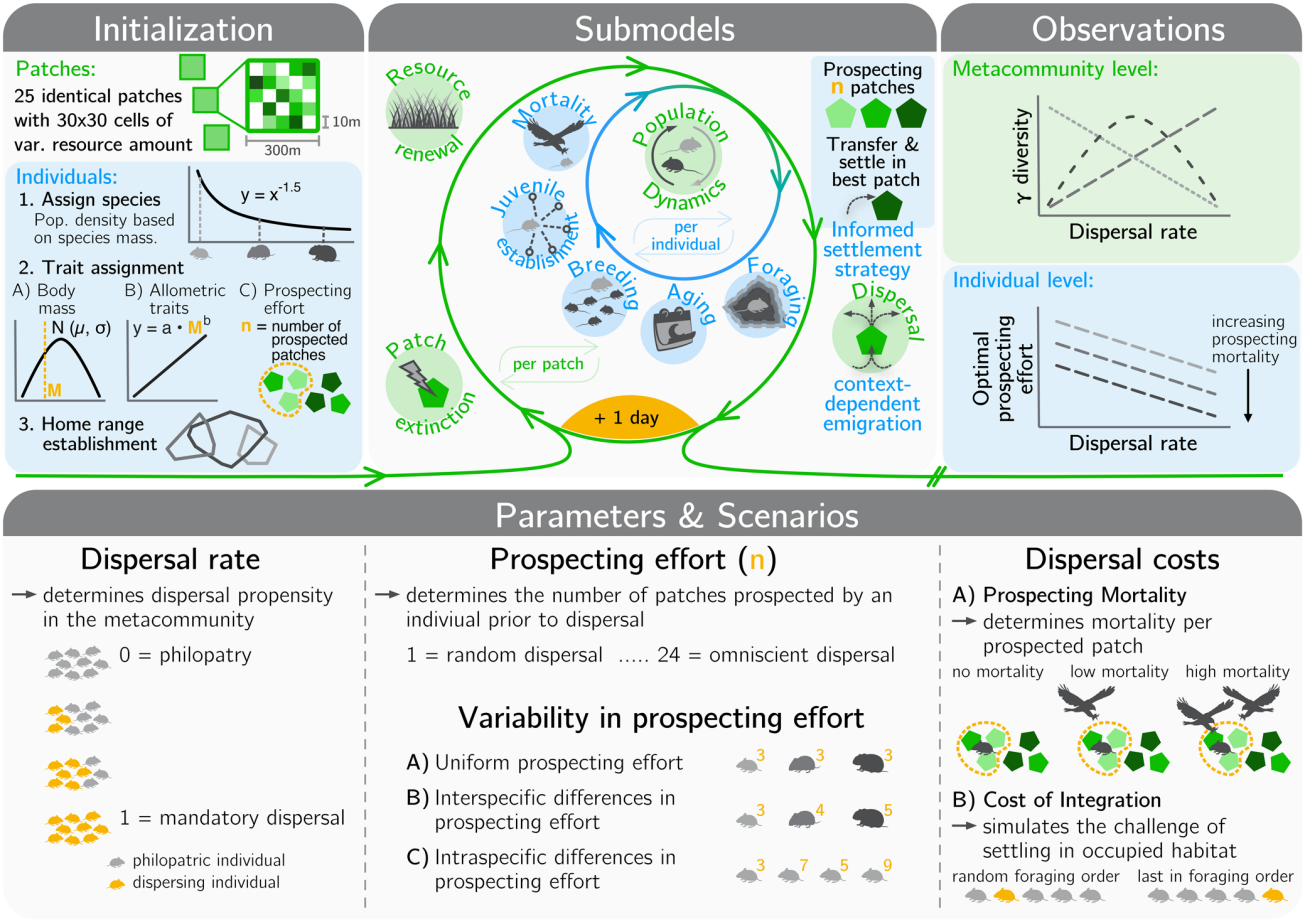
The vODD of our metacommunity model shows the initialization of the 25 patches and their local communities, the daily submodel routines for all patches and the individuals they contain, starting with random patch extinction, followed by the daily resource renewal and population dynamics in each patch. This includes the foraging behaviour of the individuals leading to resource competition, their reproduction and mortality. The dispersal process between the patches is based on an informed dispersal strategy displayed by dispersers, which prospect *n* patches and subsequently transfer to and settle in the best patch. The model output evaluates the metacommunity diversity over the full range of dispersal rates and for several different scenarios of prospecting and dispersal costs depicted in the grey box “Scenarios”.

We have developed an individual‐based metacommunity model of competing small mammal species in which we combine two fundamental community processes at different spatial scales: Resource competition at the local scale and breeding dispersal at the regional scale. The metacommunity consists of M discrete and identical habitat patches (M = 25). Habitat quality in terms of resource availability is determined by the daily density of consumers, which results from local population dynamics as well as emigration and immigration processes. The general principles and processes of the IBM are summarised below. The visual ODD (Szangolies et al. 2024) provides an overview of the main processes and outputs of the model, as well as the experimental scenarios (Figure 1). A detailed model description is available as an Overview, Design Concepts and Details (ODD) protocol (Grimm et al. 2020) in Appendix S1. The model is implemented in C++ using GCC version 12.2.0 and is available at Dryad.

### 2.1 Local Dynamics

Population dynamics in the 25 local communities are simulated in daily time steps and are based on the allometric individual‐based community model described in Rohwäder and Jeltsch (2022) and Rohwäder et al. (2024). Each of the 25 patches consists of a spatially explicit landscape grid divided into 30 × 30 grid cells of 10 × 10 m each with variable resource amount. Each community is initially composed of 10 theoretical species, inspired by the ecology of herbivorous small mammal species. The species are distinguished by their mean body mass, ranging from 10 to 100 g, and each individual receives a unique body mass from its species‐specific normal distribution (SD = 0.2*mean). The parameterisation of all physiological and life‐history traits follows an allometric approach (see Table S1.2 in Appendix S1).

Patches in the metacommunity undergo a random series of local extinctions at a similar rate (*pE*_day_ = 0.002). After extinction, the patch can be recolonised by dispersers. Resources in each patch are replenished daily and then depleted by the foraging activities of consumers. We assume complete niche overlap, where all species are functionally equivalent in their habitat requirements and resource use. Thus, no niche partitioning or trade‐offs in resource acquisition are present in the model. Individuals forage in a randomized daily order, thus modelling the competition for resource competition as scramble competition. Because each animal exploits only a body‐mass‐dependent fraction of a cell's resources (Buchmann et al. 2011), those foraging later in the sequence encounter depleted resources, indirectly reflecting resource competition in overlapping home ranges. Individuals exhibit spatially explicit, central‐place foraging behaviour. Starting from a random habitat cell (e.g., a burrow), individuals sequentially add neighbouring cells to their home range in order of proximity. This expansion continues until the individual's daily energy needs—inclusive of allometric locomotion costs (Calder 1996)—are met, or an allometric maximum home range area is reached (Kelt and Van Vuren 2001). This allows for high plasticity in home range sizes: if resources are scarce due to competition or low quality, individuals dynamically expand their home range area day‐to‐day to include new cells, provided they remain within the maximum allowable distance. The specific foraging process is detailed in the ODD (Appendix S1).

In addition to resource competition, population dynamics are driven by reproduction and mortality events. Individuals may die of senescence, based on an allometric life span (Hamilton et al. 2011), or starve if they repeatedly fail to acquire sufficient resources within their home range. Furthermore, local populations are regulated by negative density‐dependent mortality, representing mechanisms not explicitly considered in the model, such as predation, parasites or social regulation (Hörnfeldt 1994; Oli 2019; Saitoh et al. 1999). Reproduction is simulated deterministically, with females that reach maturity becoming pregnant. This reflects the high reproductive potential of many small mammals, which are characterised by high conception rates, early maturity and short gestation periods with successive litters (Golley et al. 1975; Hamilton 1949). Due to the high reproductive potential assumed in the model, we have decided not to take Allee effects into account. However, Allee effects in connection with dispersal can become important in metapopulations and metacommunities, particularly when rare long‐range dispersal events lead to low population densities and thus to mate limitation in more distant habitat patches (Banks et al. 2024; Pires and Queirós 2019). Upon reaching independence, juveniles establish their own home ranges within their natal patch.

### 2.2 Dispersal Dynamics and Prospecting Behaviour

Although the present study is based on an established small mammal community model, its aim is to produce general theoretical results oriented towards vertebrates. Due to the limited data on small mammal prospecting (Mabry and Stamps 2008), the model's assumptions about dispersal and prospecting are based on general observations of vertebrates, as well as previous modelling approaches in this area (Bocedi et al. 2014; Bonte et al. 2012; Delgado et al. 2014; Hovestadt and Poethke 2006; Ponchon et al. 2021).

Exploration (prospecting) and dispersal behaviour are modelled as spatially implicit processes. The number of daily dispersers depends on the assumed dispersal rate. Each day, a random subset of adult individuals (excluding currently lactating females) disperses to other habitat patches (results for natal dispersal are explored in Appendix S2: Figure S2.3). The emigration process is context‐dependent, that is, poor habitat patches feature a higher dispersal propensity than good quality patches. Thus, individuals from a poor patch are more likely to emigrate than individuals from a good patch. Once an individual has emigrated, it chooses a new patch to settle and establish its home range. The settlement decision is driven by the information collected during prospection, which is implemented as a ‘best‐of‐n’ strategy (sensu Ponchon 2024; Ponchon et al. 2021). In this process, a disperser gathers information on habitat quality from n unique, randomly selected patches across the metacommunity (excluding its patch of origin). The individual then deterministically settles in the single best patch among those n sampled options. While *n* = *M*−1 would simulate a disperser with perfect information, settling in the best available patch, *n* = 1 corresponds to random settlement, where the disperser settles on the first random patch encountered in the metacommunity.

### 2.3 Model Scenarios

#### 2.3.1 Dispersal Rate

The dispersal propensity in the community can vary from 0 (no dispersal/philopatry) to 1 (mandatory dispersal). This represents the dispersal propensity experienced by each individual throughout its lifetime. As we simulated daily time steps, we transformed the dispersal propensity for each species based on its average life expectancy (*e*_0_) during the simulations from a lifetime dispersal propensity (*pD*_life_) to a daily dispersal propensity (*pD*_day_) based on the following equation:

$${\mathrm{pD}}_{\mathrm{day}}=1.0-{\left(1.0-{\mathrm{pD}}_{\text{life}}\right)}^{\frac{1}{e_{0}}}$$

with: ${\mathrm{pD}}_{\mathrm{day}}$ = daily dispersal probability; ${\mathrm{pD}}_{\text{life}}$ = lifetime dispersal probability; $e_{0}$ = species average life expectancy.

Based on this calculation, it is possible, albeit unlikely, for an individual to disperse again after settling in a new habitat. The same may also occur in real communities, albeit rarely (Fuirst et al. 2021).

#### 2.3.2 Prospecting Effort

We devised three scenarios to distribute prospecting effort across individuals in the metacommunity and to account for differences in their effort to prospect for future breeding sites.

##### 2.3.2.1 Uniform Prospecting Effort

For a uniform prospecting effort, each individual in the metacommunity samples its environment with the same effort, that is the number of prospected patches n is equal for all dispersers. We varied n for the whole community from 1, which models random settlement, to *M*−1 (= 24) patches, which corresponds to complete knowledge of all patches, that is, fully informed settlement.

##### 2.3.2.2 Interspecific Differences in Prospecting Effort

We implemented a species‐specific prospecting effort by correlating this trait with the mean body mass of a species. Increasing body size is associated with longer movement distances and higher perceptual range (Kelt and Van Vuren 2001; Mech and Zollner 2002; Sutherland et al. 2000). Both allometrically scaled traits will increase a roaming animal's number of encountered and therefore prospected patches. Thus, according to these scaling laws, larger species are assumed to have a higher prospecting effort than smaller species.

##### 2.3.2.3 Intraspecific Differences in Prospecting Effort

It has been documented that even within the same species prospecting effort varies substantially between individuals (Fielding et al. 2023; Kralj et al. 2023). The variability between individuals is likely connected to personality‐dependent differences in exploration and movement behaviour (Kurvers et al. 2010; Martins et al. 2012). We implemented intraspecific differences in prospecting effort in two different ways. Prospecting effort can either vary randomly between individuals, with each individual receiving a prospecting effort between 1 and *M*−1, randomly drawn from a uniform distribution. The second scenario considers prospecting effort as a heritable trait, which is inherited from the mother with a certain variation around the parental trait.

#### 2.3.3 Costs

Dispersal involves multiple costs to dispersing individuals at all stages of dispersal: Costs of acquiring information (emigration and transfer phase); costs of movements (transfer phase); and costs of integration (settlement phase) (Bonte et al. 2012; Travis et al. 2012).

##### 2.3.3.1 Prospecting (Information Gathering) and Movement

Prospecting individuals invest effort in searching for suitable habitat. This carries a risk, as it may expose them to predators or reduce their energy reserves (Delgado et al. 2014; Suh et al. 2023). Our model reflects this by incorporating a mortality risk that increases linearly with the number of potential habitat patches explored. We consider three levels of prospecting mortality: no mortality, low mortality (0.01 probability per patch) and high mortality (0.02). Similarly, moving to a new location also carries risks. While not explicitly modelled, the prospecting mortality can be seen as a proxy for the dangers of movement, indirectly reflecting this cost. Even random settlement involves some inherent risk equivalent to prospecting a single patch.

##### 2.3.3.2 Integration

Successfully establishing oneself in a new habitat presents its own set of challenges. Among others, competition for resources with existing residents can be a major hurdle leading to density‐dependent starvation among new settlers (Olafsson et al. 1995). For example, common lizards settling in already occupied areas experienced lower growth and maturation rates compared to those finding unoccupied territories (Le Galliard et al. 2005) and after introduction showed a higher mortality than resident lizards (Massot et al. 1994). The model captures the challenges of settling in crowded environments by simulating disperser establishment only after resident individuals have completed their foraging activities. This approach effectively represents the heightened competition and limited resource availability faced by new arrivals in densely populated habitats.

### 2.4 Model Analysis

By combining the different dispersal scenarios, we explored the effect of different informed dispersal strategies on the overall diversity and structure of the metacommunity, as well as their impact on recolonisation dynamics. For this, we evaluated diversity in the metacommunity defined as the number of effective species (ENS), reflecting a robust measure of species diversity that integrates species richness and evenness (Jost 2006), which was calculated as the exponential of the Shannon entropy. Additionally, we evaluated species richness of the metacommunity as well as the species richness and diversity within a patch (α‐diversity) and the coefficient of variation in α‐diversity (Figure S2.2). We further measured the time to recolonise a patch and reassemble its small mammal community. Recolonization is measured as completed when average consumer densities have been reestablished in a disturbed patch, displaying again nearly complete resource consumption rates. Similarly, community reassembly was measured as the time it took for the extinct patch to recover its species richness to the average level of surrounding patches. Simulations were run over a period of 20 years, with 20 replications for each combination of dispersal scenarios. Additional results and scenarios, for example testing the importance of prospecting during adult dispersal relative to natal dispersal (Figure S2.3), which does affect a population's recovery capacity and time (Ponchon et al. 2015), are included in Appendix S2.

## 3 Results

### 3.1 Uniform Prospecting Effort

**FIGURE 2:**
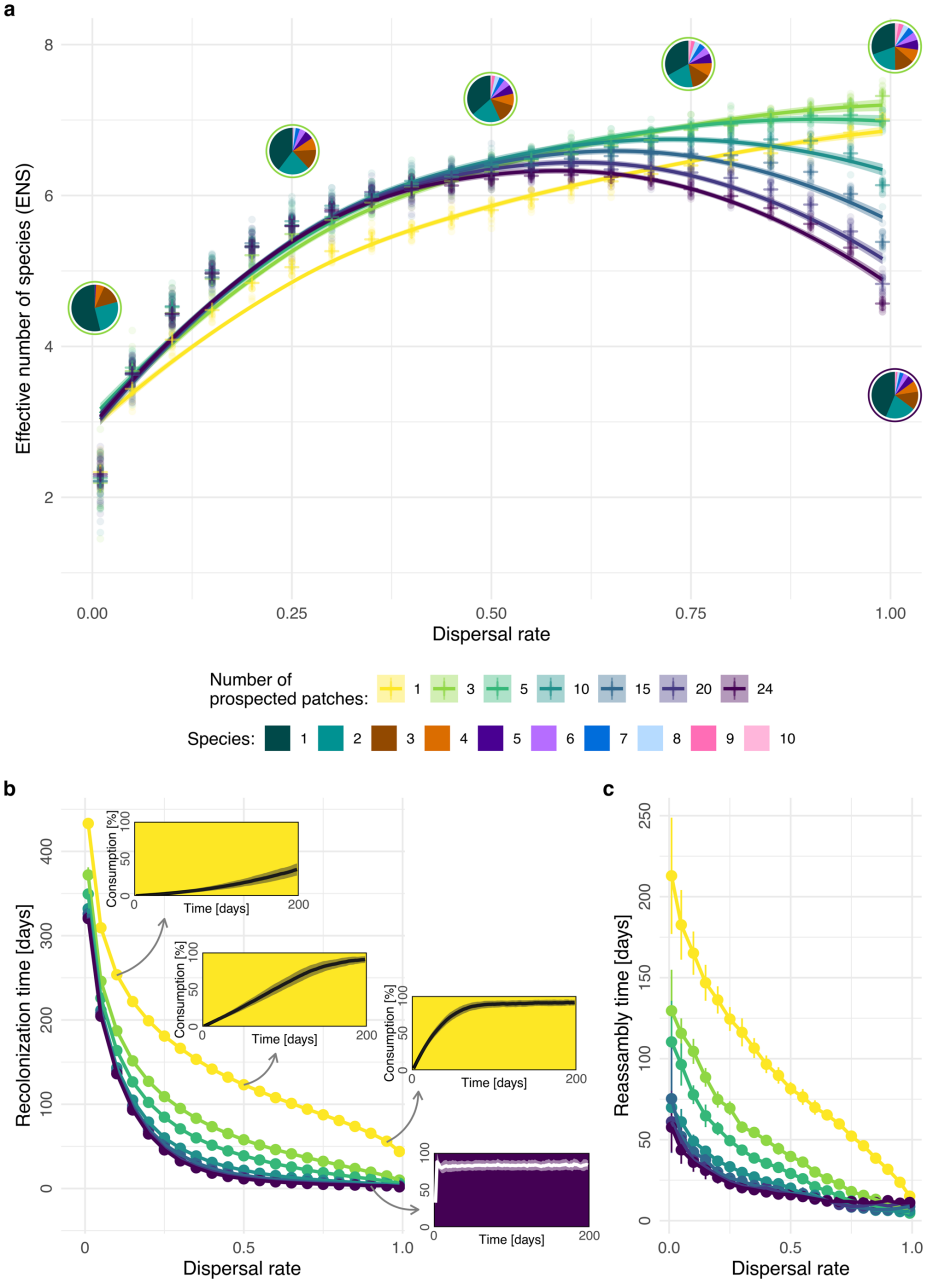
Impact of different prospecting on the dispersal‐diversity‐relationship in a competitive metacommunity (a), and the time it took an extinct patch to reach the average consumer density (b) and species richness (c) of the surrounding metacommunity patches again. The pie charts illustrate community composition for different rates of dispersal either for a prospecting effort of 3 patches (green) or 24 patches (purple). Inlay plots in b exemplify the increase in consumption in extinct patches over time due to colonization from dispersing individuals.

Informed settlement, based on the number of sites that each dispersing individual explored, had important implications for the relationship between dispersal rate and metacommunity diversity (Figure 2a). At low dispersal rates (< 0.3), both random and informed settlement increase species diversity. Yet informed settlement always outperformed random settlement. For dispersal rates greater than 0.5, settlement strategies based on 3–5 prospected patches still showed a steadily increasing effective number of species (ENS) and always maintained a higher species diversity than random settlement. On the contrary, prospecting efforts higher than 5 patches differentially penalized species diversity, with fully informed settlement being the worst strategy for maximizing species diversity.

**FIGURE 3:**
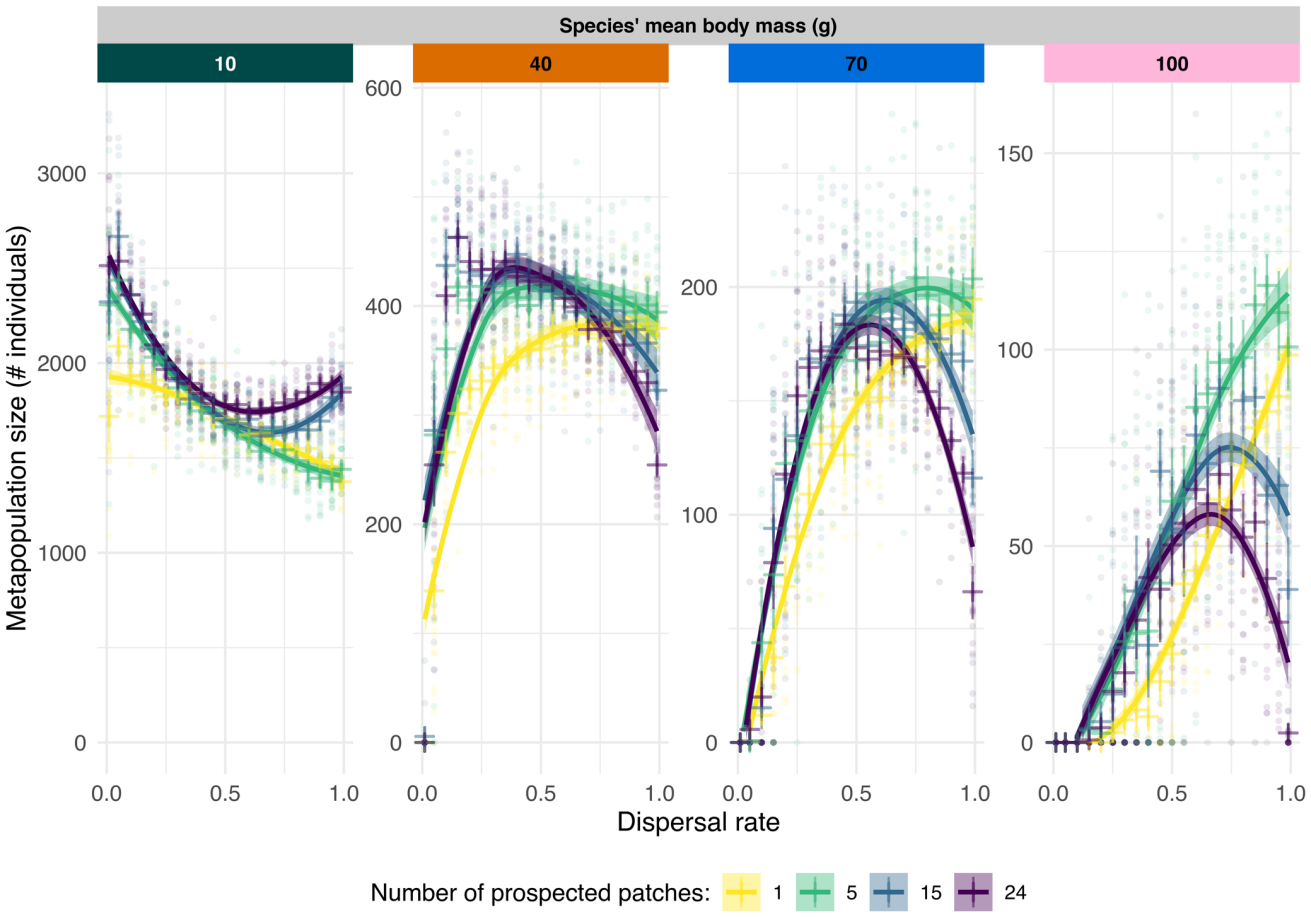
The impact of the number of patches prospected on the relationship between the dispersal rate and metapopulation size of four selected species in the simulated community after 20 years.

This pattern is also evident in the population sizes of larger species (with a mean body mass of over 40 g) (Figure 3). In contrast, the population size of the smallest species (10 g) exhibits an inverse pattern: declining population sizes at low prospecting efforts and a U‐shaped response at higher efforts.

With greater prospecting effort, a larger number of dispersers gain knowledge about the most promising location for settlement. Consequently, high‐quality resource patches attracted the highest number of immigrants, which, while increasing local competition, significantly accelerates the recovery of the community. Compared to random settlement, any level of prospecting effort substantially decreased the time required for an extinct patch to recolonize and reach its former consumer density (Figure 2b). Specifically, at low dispersal rates, a fully informed settlement strategy markedly reduces recolonization time compared to lower prospecting efforts. However, at very high dispersal rates, the difference between various prospecting strategies begins to disappear as the sheer volume of dispersers ensures that suitable patches are located regardless of prospecting effort. Furthermore, by enabling individuals to locate higher‐quality sites more efficiently, prospecting for suitable habitats also significantly accelerated the process of reassembling local communities in extinct patches (Figure 2c). However, the relative benefit of the prospecting effort depends on the overall dispersal rate. At low dispersal rates, high prospecting efforts are the most effective at restoring species diversity, while at very high dispersal rates, a slight reverse trend emerges where lower prospecting efforts marginally outperform a fully informed strategy. Despite these internal shifts, any level of informed settlement remains superior to random settlement for patch recolonization and community restoration across all scenarios.

### 3.2 Integration Costs

**FIGURE 4:**
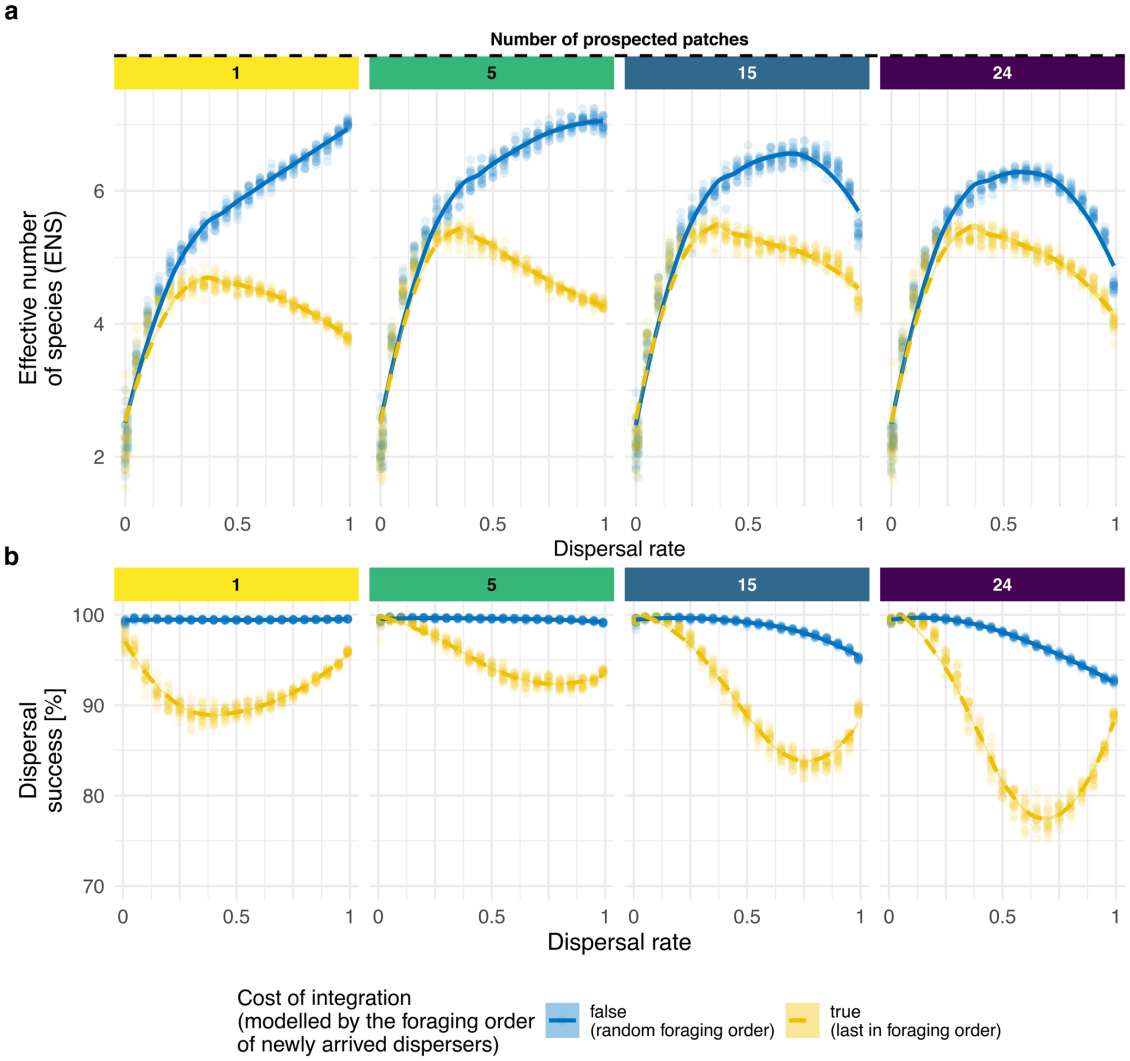
Differences in metacommunity diversity (a) and dispersal success (b) between scenarios with and without the additional cost of integration, which forces dispersers to establish in their settlement patch only after the current inhabitants foraged, causing reduced resource availability. Results are displayed for four different prospecting from random settlement (*n* = 1) to fully informed settlement (*n* = 24).

Introducing additional costs during settlement for dispersing individuals in the metacommunity has strong negative consequences for metacommunity diversity. This effect is caused by the costs associated with integrating into a new, partially occupied patch. Compared to scenarios without integration costs, gamma diversity is lower and exhibits a hump‐shaped relationship with dispersal rates (a). Random settlement, where dispersers do not actively search for suitable patches (i.e., only one prospected patch), experienced the most pronounced decline in diversity under integration costs (Figure 4a left panel: difference between yellow and blue line), while it appears that prospecting by dispersers (i.e., the number of prospected patches is five or larger) can partially mitigate this diversity loss, although only slightly.

In the absence of additional dispersal costs, metacommunity dispersal success, quantified by the proportion of dispersers that successfully colonise new patches, is generally very high, close to perfect (Figure 4b). Slightly reduced dispersal success rates only occurred for scenarios of a high prospecting effort (close to a fully informed settlement) and with high dispersal rates.

As expected, the simulation of integration costs reduced the success of dispersers to establish in a new patch (Figure 4b). Interestingly, dispersal success exhibits a non‐monotonic relationship with dispersal rates. Initially, success declines as more individuals disperse, likely due to increased competition for limited resources in existing patches. However, at very high dispersal rates, success starts to increase again. This might be explained by the availability of more vacant home ranges when a larger proportion of the individuals emigrate.

When the effects are disaggregated by species, it becomes apparent that larger species are more vulnerable to increased competition among immigrants, and in particular to the costs associated with integration (Figure S2.1 in Appendix S2). Larger species show a stronger response of their dispersal success to the additional costs of integration compared to smaller species (Figure S2.1a). Females of larger species further experienced a strong decline in their lifetime reproductive success when dispersers face the extra challenges of establishment (Figure S2.1b). A high prospecting effort can slightly offset this disadvantage.

### 3.3 Inter‐ and Intraspecific Differences in Prospecting Effort and Prospecting Mortality

**FIGURE 5:**
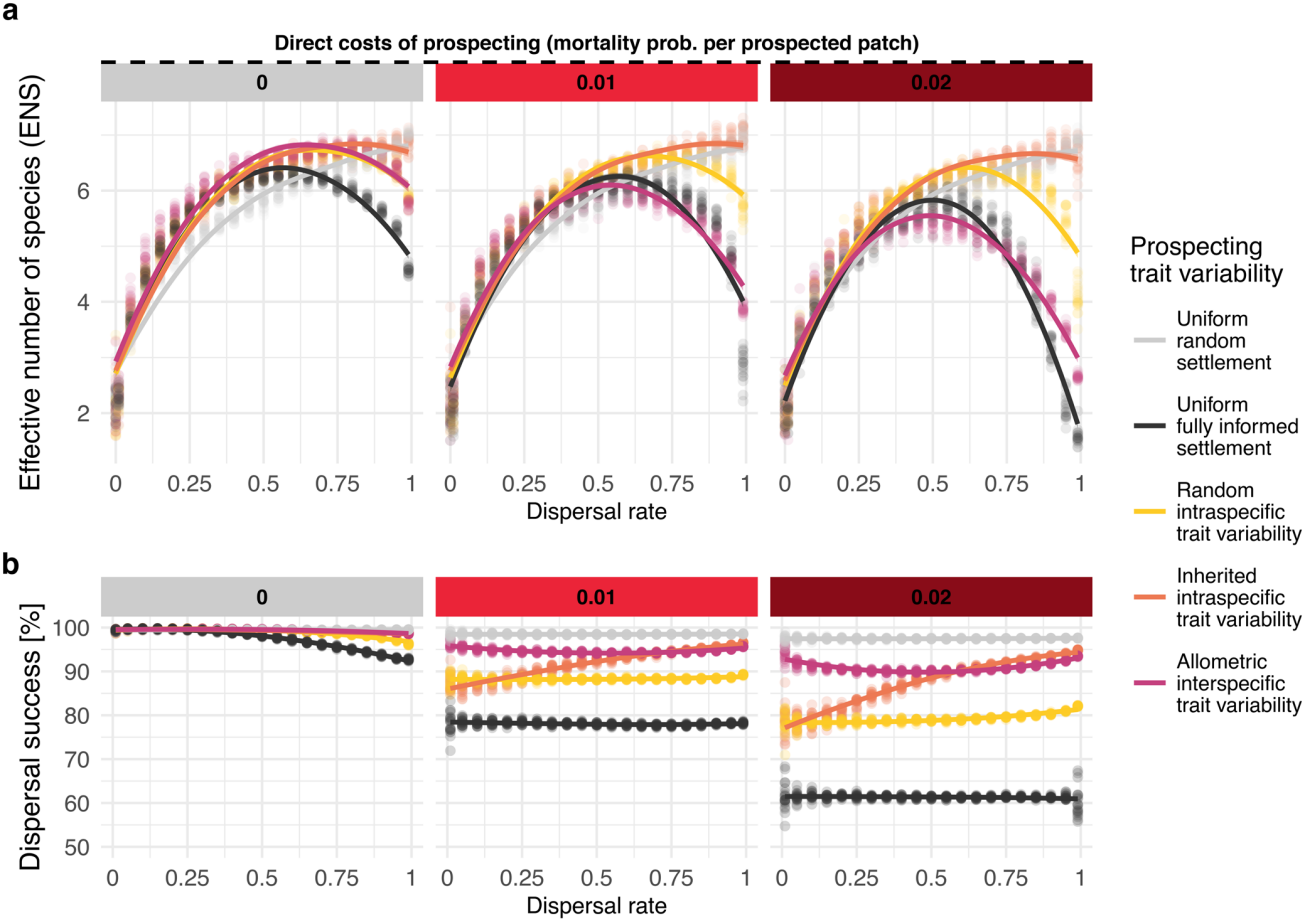
Impact of increasing prospecting mortality on the dispersal‐diversity‐relationship for different scenarios of a prospecting trait variability (a) and corresponding dispersal success in the metacommunity (b).

Figure 5 explores how different distributions of prospecting effort—ranging from species‐specific to individual‐level variation—shape the dispersal‐diversity relationship. We compare three primary scenarios: species‐specific prospecting (correlated with body mass), random individual variation (independent of species) and heritable individual variation. These are evaluated against the baseline scenarios of uniform random settlement and uniform fully informed settlement, while also accounting for the increasing mortality costs associated with prospecting.

The scenario of a species‐specific prospecting effort, positively correlated with mean species body mass, resulted in a hump‐shaped relationship between dispersal rate and gamma diversity (Figure 5a). This scenario differs fundamentally from those explored above where all species share an identical prospecting effort, as it introduces a size‐dependent trade‐off between competition and colonization.

This trade‐off is most effective at intermediate dispersal rates, where it facilitates species co‐occurrence. At low dispersal rates, smaller, competitively superior species dominate the metacommunity because larger species cannot effectively leverage their prospecting advantage for colonization. At high dispersal rates the metacommunity becomes homogenized, diminishing the benefits of prospecting and selecting the best patch. Additionally, the potential mortality risk associated with prospecting had a differential impact on competing species. Our simulations demonstrated that increasing prospecting mortality risk significantly reduces metacommunity diversity (Figure 5a), disproportionately affecting species with higher prospecting rates (i.e., larger species). This effect is most pronounced at high dispersal levels.

Interestingly, metacommunities where prospecting effort varies randomly among individuals (independent of species identity, scenario: ‘random intraspecific trait variability’) exhibited a similar, mildly hump‐shaped response of diversity to increasing dispersal rates, if prospecting incurred no additional mortality risk (Figure 5a). While this scenario was consistently outperformed by heritable variation in terms of diversity, it displayed a greater resilience to mortality risk than the species‐specific scenario (Figure 5a). Because the additional risk is distributed equally across all species rather than targeting specific functional groups (like large‐bodied species), the community structure remains more stable under high‐cost conditions.

The inclusion of a heritable prospecting effort resulted in the highest overall diversity across the dispersal gradient (Figure 5a). Remarkably, this is the only scenario that displays no diversity loss as prospecting mortality increases, and it nearly always outperforms random settlement, except under conditions of very high dispersal rates combined with high prospecting mortality (Figure 5a).

**FIGURE 6:**
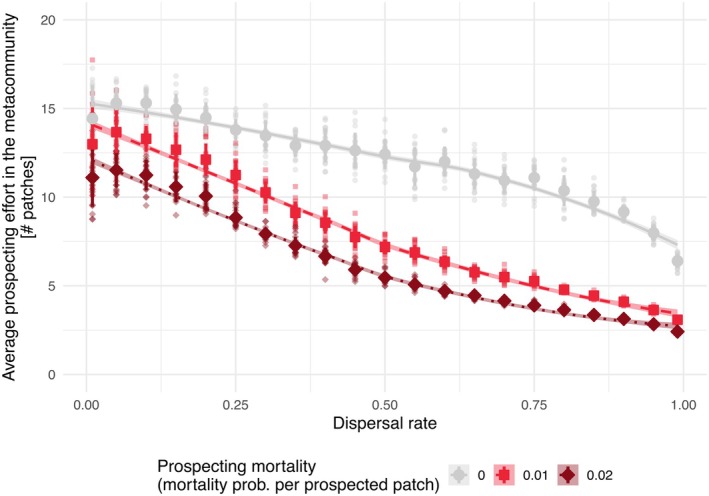
For scenarios of a heritable prospecting effort, the average prospecting effort in the metacommunity is decreased with increasing dispersal rates as well as increased prospecting mortality.

Across all scenarios, random settlement consistently yields the highest dispersal success (Figure 5b) because it avoids all prospecting‐associated costs. In contrast, fully informed settlement consistently shows the lowest success, with the negative impact scaling directly with the level of mortality risk. While dispersal rate itself has a negligible effect on dispersal success in most scenarios, the heritable scenario is the notable exception. In this case, a heritable prospecting effort led to increasing dispersal success with dispersal rate (Figure 5b). As a result of this adaptive trait response, the average prospecting effort within the metacommunity declined with dispersal rate and further decreased with increasing mortality risk (Figure 6).

## 4 Discussion

In this study, we developed an individual‐based competitive metacommunity model to investigate the role of informed dispersal in shaping the relationship between metacommunity diversity and dispersal rate. By simulating different prospecting efforts and within and between species strategies, we gained insights into how these behaviours can influence competitive species interactions and community composition across multiple patches. Our study supports Schmidt et al.'s (2010) hypothesis that species co‐occurrence or competitive displacement can be mediated through information.

### 4.1 Uniform Prospecting Effort

Our results highlight the complex interplay between informed dispersal and metacommunity diversity. For random dispersal and low prospecting rates, we observed a steady increase in diversity with increasing metacommunity dispersal rates. However, while low levels of prospecting can enhance metacommunity diversity by increasing settlement success, contrary to our initial hypothesis, higher levels of information acquisition can lead to decreased diversity. This phenomenon arises from the rapid colonization of high‐quality patches by a large number of informed dispersers, resulting in intense competition and reduced establishment success, particularly affecting species with high resource demands (see Figure 3). When dispersers with perfect information converge on the best patch, species with high resource demands and hence large home range requirements are particularly vulnerable to competitive exclusion, leading to biodiversity loss.

In well informed dispersal systems, empty patches act as a kind of ‘ecological trap’ (Robertson and Hutto 2006), attracting a high number of dispersers while thereby creating highly competitive environments and reducing habitat quality. Consequently, the predictability of the information gathered is rapidly compromised. While prospecting is often considered advantageous in predictable environments (Bocedi et al. 2012; Boulinier and Danchin 1997), complete knowledge for all individuals will rapidly erode the resource availability in the best patch, especially when disperser numbers are high. Similarly, Schmidt et al. (2010) demonstrated that occupancy rates of the highest quality patches increased with increasing prospecting effort. This rapid recolonisation of disturbed patches is thus a factor that will limit the duration of information gained by affecting the temporal autocorrelation of resource availability.

Those findings align with previous studies simulating metapopulation dynamics, which have also shown that excessive prospecting can lead to adverse density‐dependent effects (Delgado et al. 2014; Ponchon et al. 2021). For example, Ponchon et al. (2021) concluded that under perfect information gathering and use, dispersers would all cluster on the same best patch and the resulting overcrowding of good patches did drastically affect subsequent breeding success. Our study extends those insights into population dynamics, revealing effects of prospecting effort on species interactions. In our community model, examining the individual populations reveals that the population size of the heavier species conforms to the observed hump‐shaped diversity pattern (see Figure 3). However, it is interesting to note that the lightest and most abundant species show the opposite pattern: slight dispersal has a negative effect on population density regardless of prospecting effort. As the species that are most successful locally and have the smallest home range requirements, abandoning established home ranges is not compensated for by successfully settling in new habitat patches. The freed‐up resources enable heavier individuals of other species with larger home ranges to successfully establish themselves in the local areas. Only when dispersal and prospecting effort are high does the negative trend for the smallest species reverse and population size increase. The negative dynamics associated with high dispersal and prospecting effort for heavier species create more spatial niches for the smallest species with lower area requirements in both the source and target patches. Because individual fitness was differentially affected by a species' competitiveness (based on its resource and area requirements), metacommunity diversity was highly sensitive to the degree of local resource competition within a patch. Our competitive metacommunity model suggests that high prospecting in all species, especially when coupled with high dispersal rates, can undermine its benefits for biodiversity. Overall, the results also demonstrate the importance of considering the community context when evaluating the expected dispersal success of a species.

### 4.2 Interspecific Differences in Prospecting Effort

The simulated metacommunity, subject to stochastic extinction events, can be placed in the paradigm of patch dynamics (Leibold et al. 2004). This paradigm emphasises the critical role of heterogeneity dynamics created by natural disturbance regimes for the maintenance of diversity. In this context, species diversity is strongly dependent on the trade‐off between competition and colonisation. Highly competitive species may dominate specific patches, but struggle to disperse, thus hindering their ability to reach new habitats. The ability to locate temporally unoccupied, high‐quality sites during prospecting might strongly affect the competition‐colonization trade‐off. When multiple species compete for a single limiting resource, competitive ability is determined by the minimum resource requirement for population persistence (Tilman 1982, 1986). Consequently, due to our allometric trait‐based approach, the smallest species with the lowest resource needs is the superior competitor, potentially excluding all others in the absence of stabilizing mechanisms. To explore the role of prospecting within the competition‐colonisation trade‐off, we introduced variations in prospecting effort among species, linked to their average body size. Larger species, despite their inferior competitive ability due to higher resource requirements, can potentially exploit high‐quality patches through increased prospecting. Our results suggest that species with higher prospecting effort, possibly related to larger body size and greater mobility, experience increased colonisation success within the metacommunity at intermediate dispersal rates. However, when dispersal rates are increasing the advantages of prospecting are largely diminished, because high numbers of disperser will reach high quality patches even with a random settlement strategy. Furthermore, species‐specific prospecting efforts led to a significant decline in diversity when prospecting was associated with a mortality risk. This is because the costs were distributed unequally: larger species, which showed a higher prospecting effort, were disproportionately affected by increased mortality, while smaller species with lower prospecting efforts remained relatively shielded from these costs. This highlights how specific behaviours can influence a species' ability to disperse in a dynamic landscape. Depending on the environmental conditions, a species' reliance on prospecting for potential target patches can positively or negatively affect its dispersal success. On the one hand, prospecting can enable a species to locate and settle in high‐quality habitats. For example, prospecting flying squirrels were very effective at finding their preferred habitat in fragmented landscapes (Selonen and Hanski 2012). On the other hand, scenarios of increased prospecting costs show that prospecting is also a behavioural trait that can make a species particularly vulnerable to anthropogenic disturbance and landscape change. The role of the matrix in movement ecology and hence, biodiversity dynamics, has been widely highlighted (Driscoll et al. 2013; Franklin and Lindenmayer 2009; Kuefler et al. 2010; Ricketts 2001), and will also strongly influence prospecting efforts. Our model allows us to simulate these effects in a community context, showing their strong influence on biodiversity patterns.

### 4.3 Intraspecific Differences in Prospecting Effort

Empirical evidence would suggest that individuals often prospect only a very limited number of patches of their surrounding environment. Studies on seabirds consistently show that prospecting patterns typically involve one to eight different colonies (Campioni et al. 2017; Kralj et al. 2023; Oro et al. 2021). Such evidence indicates that individuals generally limit their prospecting efforts to a few nearby colonies. In accordance with this, our simulations of heritable prospecting effort did also demonstrate a decline in average prospecting effort with increasing dispersal rates as well as increasing prospecting costs, converging on an average of 2–7 prospected patches. These findings also align with predictions from eco‐evolutionary models, which demonstrate that selection favours individuals adopting a limited number of prospected patches and rarely favours the acquisition of high precision information.

Interestingly, the scenario of inherited intraspecific differences resulted in highly diverse metacommunities with comparable and even higher levels of biodiversity as the scenario of interspecific differences for high dispersal rates. Moreover, these metacommunities exhibited remarkable resilience to increasing prospecting mortality, highlighting the significant role of heritable intraspecific variation in movement behaviour. Given that dispersal‐related traits have been identified as having some of the highest average heritability among animal behaviours (Dochtermann et al. 2019), it is highly probable that prospecting, as a core component of the informed dispersal process, is subject to similar genetic underpinnings (Saastamoinen et al. 2018). This finding underscores the importance of individual variation in shaping species' responses to changing environmental conditions and suggests that promoting intraspecific diversity can enhance ecosystem resilience (Des Roches et al. 2018, 2021).

### 4.4 Perspective

At first glance, the modelled ‘best‐of‐n strategy’, which has also been used in previous modelling studies (Ponchon et al. 2021; Ponchon and Travis 2022), appears to contradict the findings of earlier mathematical models. These models indicated that an ideal free distribution could be a stable evolutionary outcome in spatially structured populations competing for shared resources (Cantrell et al. 2007, 2021; Cantrell and Cosner 2018; Netz et al. 2023). In these models, dispersal typically evolves in a way that maximizes fitness (Cantrell et al. 2007). However, there are several important differences between our simulation model and these theoretical models, including a community‐level approach, settlement prospecting and stochastic patch extinctions. This makes a direct comparison difficult. Nevertheless, studying evolutionary stable strategies and possible ideal free distributions would be a fascinating theoretical endeavour in the future. In fact, we believe that our ‘best‐of‐n’ approach, in which prospecting effort is considered a heritable trait, optimises fitness and thus moves in the direction of this area of research.

The dispersal process is a complex interplay of abiotic conditions as well as biotic factors like density, predation risk and individual phenotype (Benton and Bowler 2012; Khattar et al. 2024; Thierry et al. 2024). While our model has begun to explore some of these factors, many opportunities remain.

Recent theoretical and experimental studies have attempted to disentangle the different cues for dispersal and thereby gain a better understanding of how ecological selection influences different dispersal strategies (Khattar et al. 2024; Thierry et al. 2024). As Khattar et al. (2024) shows, landscape features strongly influence ecological selection on dispersal traits and shape the dominant dispersal strategy, which in turn will likely influence the dispersal‐biodiversity relationship.

Investigating these abiotic effects will require a more realistic parameterisation of metacommunity landscapes (e.g., incorporating features such as seasonality or spatio‐temporal heterogeneity in habitat quality). Making the dispersal process and the connected prospecting strategy spatially explicit could be used to introduce proximity and connectivity between metacommunity patches. The model, which has already been used to investigate effects of fragmentation on foraging success and local biodiversity (Rohwäder et al. 2024; Rohwäder and Jeltsch 2022; Szangolies et al. 2022), could also be used to observe effects of fragmentation at the dispersal scale on information gathering for dispersal decisions, a process for which movement ability and perceptual range would be highly relevant. As discussed above, prospecting behaviour could enhance a species' success to locate preferred habitats in fragmented landscapes but could also make a species more vulnerable to adverse conditions in the matrix as it is for dispersal (Stamps et al. 2005; Yamaura et al. 2022). A spatially explicit approach to modelling patches in the metacommunity would also allow different regimes of spatiotemporal autocorrelation to be incorporated not only in the overall habitat quality of patches but also in their disturbance regime.

Furthermore, how animals use environmental cues is determined by how they perceive and integrate information about their environment (Benton and Bowler 2012; Enfjäll and Leimar 2009). An organism will integrate not only abiotic habitat conditions into its decisions, but also biotic information about intra‐ and interspecific competition. As Thierry et al. (2024) have shown experimentally, dispersal rates depend on temperature as well as the presence and density of other species. Therefore, some individuals may choose sup‐optimal habitat conditions to avoid highly competitive environments, which could lead to niche partitioning between dispersal and habitat selection strategies.

Our flexible modelling framework allows us to easily incorporate further dependencies and availabilities of information into decisions at all stages of the dispersal process. For instance, implementing a state‐dependent emigration, where individuals' decisions are influenced by their internal state, such as energy reserves or past breeding success (Oro et al. 2021; Ponchon et al. 2017). This would extend the decision‐making process to include public as well as personal information and can provide valuable insights into how individual variation shapes community dynamics. Additionally, expanding upon our modelling of the effect of phenotype‐dependent dispersal (see Figure S2.4 in Appendix S2) to incorporate covariation with other traits, like competitiveness or aggressiveness (Duckworth and Kruuk 2009), could reveal how these interactions influence community structure. For example, in some rodent species, a competitive advantage in territorial contests has been observed to be conferred by larger body size (Luque‐Larena et al. 2003; Schüler and Renne 1988).

In this study, we first consider the effects of variability at the interspecific and intraspecific levels separately, in order to reveal possible effects and the underlying mechanisms. It is, of course, to be expected that both sources of variability occur simultaneously in real systems. Investigating this increased complexity in more detail would be another interesting next step for future studies.

By further developing the model to account for these complexities, we can gain a deeper understanding of the mechanisms driving metacommunity dynamics and the role of prospecting in shaping biodiversity. We acknowledge prospecting (or habitat selection) is a trait under ecological selection, and its influence on the dispersal‐biodiversity will likely depend on the type of competitive dynamics and landscape characteristics, as suggested by Khattar et al. (2024) and Thierry et al. (2024).

## 5 Conclusion

Our individual‐based metacommunity study highlights how prospecting for informed settlement decisions can affect the relationship between diversity and dispersal in a metacommunity context. The degree of information acquisition influences competitive species interactions and co‐occurrence, as well as recolonisation rates and resilience to environmental change. The study showed that neither random nor fully informed settlement is most beneficial for biodiversity. Instead, a low prospecting effort maintained the most diverse communities, balancing the negative consequences of intense competition for the best patches and suboptimal habitat selection.

## Author Contributions

**Marie‐Sophie Rohwäder:** conceptualization (lead), data curation (lead), formal analysis (lead), investigation (lead), methodology (lead), software (lead), validation (lead), visualization (lead), writing – original draft (lead). **Florian Jeltsch:** conceptualization (supporting), data curation (supporting), formal analysis (supporting), funding acquisition (lead), investigation (supporting), methodology (supporting), project administration (lead), software (supporting), supervision (lead), validation (supporting), visualization (supporting), writing – original draft (supporting).

## Funding

This work was financed by the German Research Foundation (DFG) in the framework of the BioMove Research Training Group (DFG‐GRK 2118/1). Open access funding enabled and organized by Project DEAL.

## Conflicts of Interest

The authors declare no conflicts of interest.

## Supporting information


**Table S1.1:** State variables of landscape cells.
**Table S1.2:** State variables of individuals.
**Figure S1.1:** Flow chart illustrating the scheduling of the model's main processes and the entity executing them.
**Figure S1.2:** (A) displays relationship between lifetime dispersal rate and daily dispersal rate for each species. (B) shows the resulting total number of daily dispersers for each dispersal propensity scenario.
**Figure S1.3:** (A) shows the species‐specific prospecting effort for prospecting scenario B of interspecific differences. Prospecting effort is steadily increasing with the species average body mass. (B) shows the resulting correlation between mother and offspring trait in prospecting effort for prospecting scenario C of inherited intraspecific differences. Offspring prospecting effort varies around the maternal trait with SD = 3.
**Table S1.3:** Dispersal‐related model parameters.

**Figure S2.1:** Species reproductive success (a) and dispersal success (b) for different scenarios of prospecting effort and integration costs, showing the disproportionate impact on larger species, which have higher energy requirements and are particularly vulnerable to increasing competition.
**Figure S2.2:** Gamma (γ) and alpha (α) species richness (a, b) and effective number of species (c, d), giving a more balanced impression of species composition. Due to the reducing effect of disturbed patches, α‐diversities (b, d) are consistently slightly lower than γ‐diversities (a, c). Otherwise, α‐ and γ‐diversities show the same patterns, as we simulated identical patches and no differences in habitat requirements between species. Variability in the effective number of species (e) between patches in the metacommunity is reduced as dispersal increases, due to the homogenising effect of dispersal. Informed colonisation and settlement further reduced variability due to rapid recolonisation of disturbed patches.
**Figure S2.3:** Juvenile dispersal displays no negative effect of high prospecting effort on diversity due to the much lower number of daily dispersers. Thus, juvenile dispersers always experience less competition. Dispersal rate indicates the daily proportion of offspring that will disperse from their natal patch after reaching independence.
**Figure S2.4:** We found no impact of a phenotype‐dependent dispersal on biodiversity in our metacommunity model. For a phenotype‐dependent dispersal, dispersers were either larger (dark blue) or smaller (light blue) than their respective species' average body mass compared to dispersers being of a random body mass (grey).

## Data Availability

Data files are available from the Dryad Digital Repository: https://datadryad.org/dataset/doi:10.5061/dryad.zkh1893kt. The model software is accessible at Zenodo: https://doi.org/10.5281/zenodo.14198128.
