# Supplementary material for: How Prospecting for Informed Dispersal Shapes Biodiversity Patterns in a Metacommunity
Source: Ecol Evol. 2026 Jul 7;16(7):e73912. doi: 10.1002/ece3.73912 (PMC13341221; doi:10.1002/ece3.73912)
Supplement: Supplementary file 1 — Table S1.1: State variables of landscape cells. Table S1.2: State variables of individuals. Figure S1.1: Flow chart illustrating the scheduling of the model's main processes and the entity executing them. Figure S1.2: (A) displays relationship between lifetime dispersal rate and daily dispersal rate for each species. (B) shows the resulting total number of daily dispersers for each dispersal propensity scenario. Figure S1.3: (A) shows the species‐specific prospecting effort for prospecting scenario B of interspecific differences. Prospecting effort is steadily increasing with the species average body mass. (B) shows the resulting correlation between mother and offspring trait in prospecting effort for prospecting scenario C of inherited intraspecific differences. Offspring prospecting effort varies around the maternal trait with SD = 3. Table S1.3: Dispersal‐related model parameters. [file ECE3-16-e73912-s002.pdf]

ODD Protocol for an allometric metacommunity model of informed dispersal

This is an ODD protocol document ("Overview, Design concepts, and Details"), which provides a detailed description of the model presented in: "How Prospecting for Informed Dispersal Shapes Biodiversity Patterns in a Metacommunity" in Ecology & Evolution

Marie-Sophie Rohwäder & Florian Jeltsch
University of Potsdam, Department of Plant Ecology and Nature Conservation, Germany

Here we present the model description following the Overview, Design concepts and Details (ODD) protocol for describing agent-based models (Grimm et al. 2006, 2010, 2020). This model combines local population dynamics and resource competition in a herbivorous small mammal community based on previous models (see Rohwäder and Jeltsch 2022 and Rohwäder et al. 2024) with dispersal dynamics in a metacommunity context. Throughout the ODD parts that connect to the new dispersal process are highlighted in orange. The model was implemented in C++ using GCC version 12.2.0 as well as Apple clang version 14.0.3. The code is open-source and can be downloaded from zenodo at https://doi.org/10.5061/dryad.zkh1893kt.

THE MODEL

Purpose and patterns

The purpose of this model is to explore the role of informed dispersal strategies for the relationship between gamma-diversity and dispersal rate in a metacommunity context of competing small mammal species. The main focus is on the process of gathering information at local and distant scales to inform context-dependent emigration and settlement choices. This is a common behaviour, known as prospecting (Reed et al. 1999), across a variety of taxa (Ponchon 2024). Prospecting plays an important role for shaping individual dispersal decision and is hence an integral part to informed dispersal, which describes the concept that individuals gather and integrate information at all stages of dispersal: emigration, transfer and settlement (Clobert et al. 2009). With our model we investigate how differences in prospecting effort (expressed as the number of patches in the metacommunity that are prospected by the dispersing individuals prior to dispersal) can shape the diversity-dispersal relationship, which is a core concept of metacommunity ecology. Prospecting effort can thereby either vary between metacommunities, species, or individuals. Biodiversity in our model emerges from two fundamental community processes at different spatial scales: Resource competition at the local patch scale and dispersal at the regional metacommunity scale.

Entities, state variables and scales

The model features three different entities: *Patches*, which consists of *landscape cells* and are inhabited by the *individuals*. The model uses discrete time steps, with a temporal resolution of one day and total timescale of 20 years (i.e., 7300 days).

The metacommunity consists of M identical *patches*, of a size of 9 ha (300 x 300 m). All patches are represented as a grid of 30 x 30 landscapes cells, which are the smallest unit of a patch, defining its spatial resolution.

While local processes like foraging are simulated as spatially explicit processes on the grid of a patch, the space between patches and hence the prospecting and dispersal processes are spatially implicit.

Landscape cells are presented as square cells, with a length and width of 10 m. They are described by their location in the patch, their habitat type and their resource amount (see Table S1.1). The model distinguishes between two habitat types: Habitat or matrix. While habitat cells provide food resources for the individuals, cells belonging to the matrix are considered non-productive. The sum of resources in each landscape cell defines the overall habitat quality of a patch. Prospecting animals will know the habitat quality of the patches they prospected, which will in turn affect their settlement decision. While all patches start with the same overall habitat quality, which is renewed daily, the resource availability is subsequently decreased by the daily foraging activities of the local consumers. The effective consumer density in each patch results from local population dynamics, emigration and immigration processes as well as stochastic extinction events.

Table S1.1: *State variables of landscape cells.*

**State variable**	**Unit**	**Description**
Resource amount	Dry biomass, $g/cell \cdot day$	Availability of food resources in one cell
Habitat type	-	Defines the habitat of the cell as either productive habitat or non-productive matrix
Location	-	Stores the x and y coordinates of the cell to define its exact location in the patch

The simulated *individuals* represent small mammals belonging to different species of a non-flying, herbivorous mammal community. The model simulates ten different, theoretical species, which are characterized by their mean body mass and a normal distribution around this mean (sd = 0.2 * mean). Individuals of each species obtain a unique body mass from their species-specific body mass distribution. For animals the body size is one of the most important and meaningful functional traits as it determines variation and constraints for other physiological as well as ecological traits (Shingleton 2010). Allometric relationships are extensively studied and well-established and are thus an adequate method to parameterize important state variables in the model. Consequently, the body mass of an individual is used to calculate most other relevant traits. This includes physiological traits such as the daily energy requirements of individuals calculated from the allometric equations of the field feeding rate for herbivores given by Nagy (2001), the locomotion cost per distance reported by Calder (1996) or the natal dispersal abilities of juveniles stated in Sutherland et al. (2000). Other life history traits like the average lifespan of an individual, its mean gestating and lactating period, mean litter size or age at maturity were taken from Hamilton et al. (2011). Individuals are further described by their age and sex. All individuals in this model are considered central place foragers, which have a strong focus on one central place in their home range, which is visited very frequently (Orians 1979). Each individual in the model uses a random location in its natal patch as its central place to form a home range. This is also referred to as the core cell of a home range, where the animal is assumed to have its den or burrow. Dispersing individuals receive a new core cell in their settlement patch to establish a new home range. Individuals now additionally display a prospecting effort (n), which expresses how many patches in the metacommunity an individual has prospected before dispersal. This new trait describes the effort an individual demonstrates to sample potential destination patches and determines the amount of information that can be used to inform settlement decisions. While $n = 1$ would resemble "blind" dispersal, in which case the disperser would just randomly choose a settlement patch, $n = M - 1$ would resemble "omniscient" dispersal, the disperser knows the habitat quality of all patches in the metacommunity that are not its current location and can hence choose the best.

Table S1.2: *State variables of individuals*

State variable	Unit	Allometric relationship	Author	Description
Species	-			Defines the species of the individual
Body mass	*kg*			Body mass of individual
Age	days			Counts the age of the individual
Sex	male/female			Sex ratio is assumed as 1:1
Prospecting effort	Patches			Prospecting effort determines the number of patches prospected prior to dispersal
Location	-			Core cell position of the individual's home range in the landscape
**Allometric traits:** [1]				
Maximum home range	ha	$56.23 \cdot M^{0.91}$	Kelt and Van Vuren (2001)	Maximum home range area used as a constraint for home range size
Feeding rate	Dry matter in g/day	$65.765 \cdot M^{0.7628}$	Nagy (2001)	Minimum amount of food resources, that need to be contained in the home range calculated as daily intake rates for dry matter in grams of food.
Food exploitation	-	$\gamma \cdot \left(\frac{M}{0.001}\right)^{-0.25}$	Buchmann et al. (2011)	Defines magnitude of food resource exploitation; $\gamma = 1$ and the denominator of 0.001 $\mathrm{kg}^{-0.25}$ was chosen to ensure that the food share factor varies between 0 and 1
Locomotion cost	J/m	$10.7 \cdot M^{0.68}$	Calder (1996)	Locomotion costs converted to grams of dry biomass for moving one cell forward
Maximum lifespan	days	$1766.53 \cdot M^{0.21}$	Hamilton et al. (2011)	Maximum lifespan
Maturity	days	$293.17 \cdot M^{0.27}$	Hamilton et al. (2011)	Age at first reproduction
Mean litter size	individuals	$2.24 \cdot M^{-0.13}$	Hamilton et al. (2011)	Average number of offspring produced at one birth
Gestation period	days	$64.14 \cdot M^{0.24}$	Hamilton et al. (2011)	Length of gestation period
Lactation period	days	$57.16 \cdot M^{0.22}$	Hamilton et al. (2011)	Length of lactation period
Fasting endurance	days	$9.3 \cdot M^{0.44}$	Lindstedt and Boyce (1985)	Survival time during a period of resource shortage
Natural mortality	-	$0.072 \cdot M^{-0.24}$	McCoy and Gillooly (2008)	Body mass dependent, temperature-corrected natural mortality rates per year for mammals

[1] The body mass M is required in the unit kg for all allometric equations.

Process overview and scheduling

Figure S1.3 illustrates the sequence of the model's main processes. Each patch within the metacommunity undergoes four primary daily submodels in this order: 1. patch extinction, 2. resource renewal, 3. population dynamics, and 4. dispersal. The last two patch submodels, population dynamics and dispersal, incorporate additional subroutines that are executed by each individual currently residing within the patch. Patch extinction is a stochastic event that occurs randomly with a daily probability of $pE = 0.0002$ for each patch, resulting in the immediate elimination of its entire consumer population.

The resource renewal submodel replenishes landscape cells with resources, differentiating between habitat and matrix cells, and ensures a consistent overall habitat quality across all patches. To simulate the population dynamics in a patch, different individual-level processes are modelled. All individuals get one day older. They forage in their home ranges and females may become pregnant or have offspring. Juveniles reaching independence disperse within their natal patch and try to establish their own home range within a natal dispersal distance of their mother's den. Each day individuals also face a certain chance to die, which can happen either due to an insufficient food supply in the home range or due to senility. Other causes of death, which happen well before an individual's maximum lifespan, such as disease, predation or accident are considered by applying a natural mortality rate for each individual, which shows a negative mass dependence (McCoy and Gillooly 2008), as well as a density-dependent mortality probability. For the dispersal process, a certain number of dispersers, depending on the overall dispersal rate (pD) in the metacommunity, is randomly selected in each patch, depending on the context-dependent emigration scenario. Dispersers deterministically transfer and settle in the best patch they have prospected, displaying a 'best-of-n' strategy in their decision-making process (sensu Ponchon 2024; Ponchon et al. 2021).

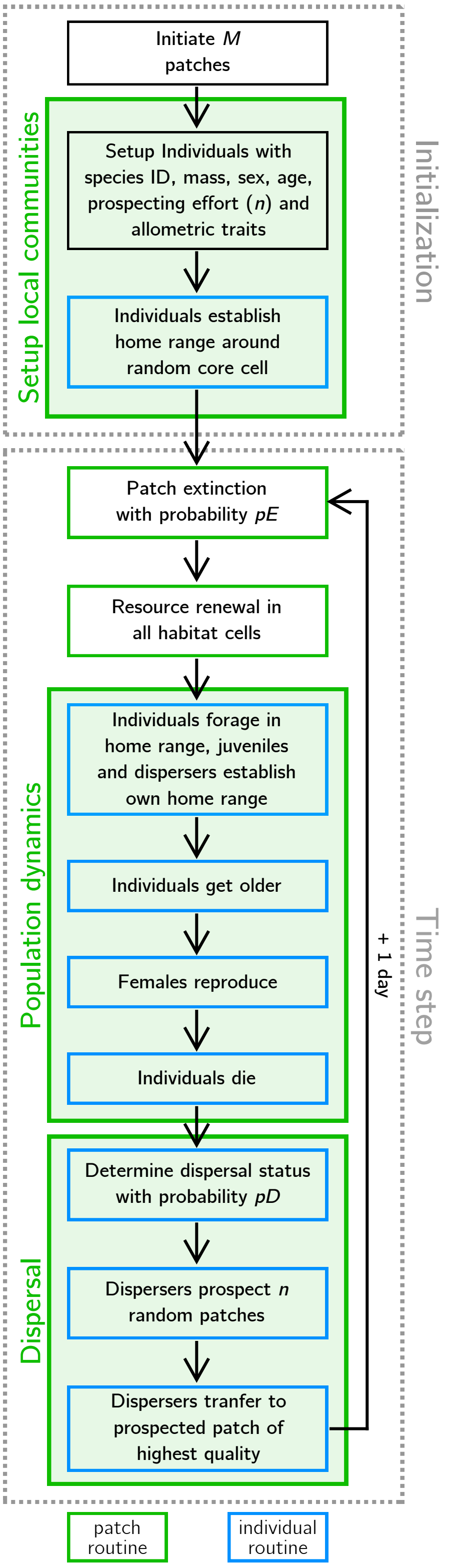

Figure S1.1: *Flow chart illustrating the scheduling of the model's main processes and the entity executing them.*

Design concepts

Basic principles

The metacommunity model simulates the dispersal and interactions of organisms competing for resources across multiple patches subject to spatio-temporal environmental disturbances. The spatially implicit dispersal is random, with a probability of pD. Dispersers use an informed dispersal strategy, sampling the habitat-quality of n random patches and, following a 'best-of-n' strategy (Ponchon 2024; Ponchon et al. 2021), choosing the highest-quality patch to establish.

Local population dynamics of all interconnected patch communities are based on a dynamic spatial home range model (Buchmann et al. 2011; Rohwäder et al. 2024; Rohwäder and Jeltsch 2022; Szangolies et al. 2022; Teckentrup et al. 2018). Allometric relationships are used to calculate various traits such as life history traits, energy requirements and locomotion costs (see Table S1.2 – Allometric traits) based on body mass.

Individual home range formation is guided by a central place foraging strategy and is controlled by food availability and physiological parameters such as daily energy demand, locomotion costs and area constraints. It follows the area-minimizing principle from optimal foraging theory (Mitchell and Powell 2004, 2012).

Emergence

Local community structure and composition emerge from the group of resident and dispersing individuals, that are able to successfully establish a home range in the landscape and reproduce. Home range establishment is driven by individual traits and food availability.

Objectives

Dispersers aim to settle in the best available patch in the metacommunity with high resource availability and low levels of competition. Therefore they choose the highest-quality patch from a random subset of n prospected patches to establish.

All individuals need to satisfy their energy requirements within the smallest possible home range following the area-minimizing-principle by Mitchell and Powell (2004). A home range can only acknowledge discrete landscape cells. Therefore, individuals add cells with the maximum food gain to their home range, which is achieved in cells with a high food availability and the shortest distance to the core cell.

Sensing

Individuals can sense the food availability and habitat type of all 8 neighboring cells around their current location. Dispersers gan knowledge of the current overall resource availability of all n prospected patches.

Interaction

Interactions between individuals arise in shared resource cell's, which belong to more than one home range. Those interactions are indirectly modelled and evolve from resource competition during home range formation and foraging. During home range formation in the setup and foraging in the established home range, an animal consumes only a share of the resources in each visited cell belonging to the home range. The rate of resource exploitation is body mass dependent and calculated using an allometric factor tested by Buchmann et al. (2011). Following individuals encounter a landscape with partly reduced food availability. This describes competition for resources in overlapping home ranges. Thus home ranges are not territorially defended by its owner.

Stochasticity

Patch extinction is a stochastic process with a daily probability of occurrence pE. Dispersing individuals are randomly assigned based on the overall dispersal propensity of the metacommunity pD, which is used to calculate daily dispersal probabilities based on a species' average lifetime (see also Equation (5)). Individual body masses are randomly drawn from their species-specific normal body mass distribution. Other stochastic processes are the selection of the home range core cell, which is randomly selected from all habitat patches in a patch.

Observation

Gamma and alpha diversities of the meta- and local patch communities are mainly expressed as the effective number of species (Jost 2006). The effective number of species (ENS) represents the number of species that are equally abundant in the community and is calculated as the exponential of Shannon entropy (Jost 2006). It is, thus, a robust measure of species diversity that integrates species richness and evenness. The model also measures a recolonization as well as reassembly time of extinct patches. Community reassembly was measured as the time it took for the extinct patch to recover species richness to the average level of surrounding patches. Further measures extracted from the simulations are consumer densities in the patches, dispersal and reproductive success, and the daily number of foragers in a habitat cell. In simulations that modelled prospecting effort as an inherited trait, the model further reported the average prospecting effort in the evolved metacommunity.

Initialization

Patches

For each simulation M patches are generated that are of the same size and overall habitat quality. Each patch features 25% habitat of intermediate habitat fragmentation. Habitat fragmentation within the patches is generated based on an algorithm that achieves a variable clumping of habitat cells through a higher probability of none-productive matrix cells turning into a productive habitat cell when they are adjoining another habitat cell during patch generation. Different levels of habitat fragmentation can be achieved by increasing or lowering this probability. An intermediate level of habitat fragmentation was used for all simulations. When the specified fraction of habitat cells is reached patch generation is terminated, leaving the remaining cells in a patch as unproductive matrix.

The food resource availability of each habitat cell is set and later renewed daily with a resource amount of 68.5 g dry biomass/day · cell, which corresponds to the overall productivity of shrub- and grasslands (Whittaker 1975). Of this total resource amount only a share of 10 % is available to the community. The remaining resources are considered unsuitable for animal consumption or lost to other taxonomic groups (Buchmann et al. 2011, 2012).

Individuals and trait assignment

Each patch assembles its own local community. Community assemblage starts with the creation of the individuals after the patches are prepared and filled with resources.

The main characteristic of an individual in the model is its body mass, which depends on the species to which the individual belongs. Therefore in a first step each individual is assigned to a species. The probability of being assigned to one of the included species depends on the mean body mass (M) of each species and is calculated from the general power-law distribution provided by Buchmann et al. (2011):

$$p(M) = \kappa \cdot M^{\eta} \quad (1)$$

In this general power-law distribution κ is a constant chosen so that the equation integrates to 1. The exponent η of the relationship determines how uniform the distribution of body masses among species is, with an exponent of 0 being the special case of a uniform distribution. The exponent for all simulations was chosen as -1.5, a value in the range tested by Buchmann et al. (2011), which yielded a realistic community structure.

This ensures that species with a lower mean body mass have a higher density of individuals in the landscape than heavier species. It thus defines the regional species pool, from which individuals can be drawn.

When an individual belongs to a species, its body mass is randomly picked from the species' normal body mass distribution and all following traits are assigned based on allometric relationships. All state variables further characterizing an animal are listed and described in Table S1.2. The sex ratio is considered 1:1 for all species but male and female individuals show no differences in their foraging behavior apart from females adapting their energy requirements during the time of gestation and lactation. Each individual is assigned a prospecting effort, which specifies the number of patches that are considered as known by the individual at the time of its dispersal. It hence determines the size of the subgroup of patches that an individual can choose from and disperse to.

All individuals are entering the landscape in succession and try to establish their home range according to their energy requirements. In the setup procedure during the first home range formation, the location of a core cell for each individual is randomly chosen and not influenced by the presence of conspecifics. As all individuals of the same species are placed independently in the landscape, the model assumes the absence of territorial behavior. During model initialization each individual has up to 100 attempts to find a suitable home range, while following juveniles, searching for a home range during the model run, and dispersers, establishing a new home range in their new settlement patch, have ten attempts. Home ranges are established around the random core cell based on the submodel *Foraging.* Thereby individuals constantly add cells to their home range starting with the closest and stepwise increasing their search radius until either their maximum home range is reached or their energy demand is fulfilled. The first acceptable home range, which can satisfy the energy needs of an individual, is taken and resources depleted. Animals not able to settle in an appropriate home range within the number of allowed attempts, will die and are excluded from the community.

Submodels

The following paragraphs summarize the functionalities of the most important procedures in the model, which shape the path and actions of the model's agents. The order of paragraphs roughly follows the schedule of the model (see Figure S1.3).

Patch extinction

The metacommunity experiences stochastic extinction events, with a probability of extinction (pE) set at 0.002. This translates to each patch facing a daily extinction risk of 0.2 %, resulting in an annual extinction probability of approximately 50 %. When an extinction event strikes a patch, all individuals currently inhabiting that patch are lost and immediately removed from the simulation. Vacated patches can only be recolonized by dispersers.

Update resources

The cell's resources are provided at a daily basis. The availability of resources within one cell is scaled to average an overall grassland productivity of 0.685 dry $\mathrm{g \cdot m^{-2} \cdot d^{-1}}$ after Whittaker (1975) of which only a fraction of 10 % is available to the simulated community.

At the beginning of each new day the resources are replenished in each productive habitat cell and all patches yield the same overall resource amount. Resources subsequently get reduced by the foraging activities of the individuals.

Local population dynamics

Population dynamics within a patch are shaped by various individual-level processes. The following subroutines, that drive these dynamics, are executed by each individual currently residing within the patch.

A) Foraging. One of the key processes of the model is the home range behaviour of all individuals. The home range is defined as the area that contains enough food resources to cover the individual's daily feeding rate and its locomotion costs, while foraging within the home range. To simplify the spatial foraging patterns of the agents, the movement can only consider discrete patch cells. An allometrically scaling maximum home range size for herbivorous mammals is used as reported by Kelt and Van Vuren (2001) to define an upper boundary for an individual's home range area.

The model assumes that all individuals are central place foragers frequently returning to a central place within their home range (Bell 1990). This is implicitly represented in the model by calculating the locomotion costs for each foraging bout as an outreaching journey and a return to the core cell using the Euclidean distance. Agents do not pay those locomotion costs when visiting a matrix cell, but are expected to continue their exploration for resources. Thus, matrix cells do not entail additional locomotion costs but still add to the movement distances of foraging in habitat cells in the periphery.

Newly created individuals, juveniles becoming independent and dispersers settling in a new patch need to establish a new home range. The search for an appropriate home range starts with the choice of a core cell, the central place of the home range. This cell is randomly chosen from the pool of all productive habitat cells in a patch.

After an animal has found a core cell, more cells are added consecutively to the home range. All animals start exploring the cells in the closest distance to their core cell and gradually increase their hame range area. It is assumed that an animal can sense the food availability of the 8 neighboring cells of its current foraging location, which determines the decision for its next foraging bout.

The net food gain NFG_i of the added cell i is calculated and compared to the minimum food requirement of the individual. NFG_i in each cell is defined as the difference between the exploited food and the locomotion costs to and from this cell. The amount of exploited food is the arithmetic product of food availability in the cell R_i and the individual magnitude of food exploitation (FE_i [see Table S1.2]).

$$NFG_i = R_i \cdot FE_i - 2 \cdot LC_i \quad (2)$$

$$\begin{aligned} \text{with:} \quad NFG_i &= \text{Net food gain in cell } i \\ R_i &= \text{Food availability in cell } i \\ FE_i &= \text{Individual magnitude of} \\ &\quad\ \text{food exploitation} \\ LC_i &= \text{Locomotion costs to cell } i \end{aligned}$$

Discrepancies were observed between the scaling of metabolic needs (exponent of 0.75) and the scaling of home range size with an exponent of around 1 (Haskell et al. 2002). Amongst others, this was explained by a coarser resource perception of large animals, whose resource exploitation is thus reduced (Haskell et al. 2002).

In the model the factor FE_i is accounting for the part of the available resources in a cell that are exploited by an individual. Accordingly it has an allometric exponent of -0.25 (see Table S1.2). Other mechanisms proposed to explain the discrepancy between the scaling of metabolic needs and home range size are discussed in Buchmann et al. 2011 but would ultimately lead to the same scaling of FE_i.

Locomotion costs LC_i are calculated allometrically for each individual as given in Calder (1996) (see also Table S1.2) and expressed in equivalent amounts of dry biomass per unit with a conversion factor for non-fermenting herbivores reported by Nagy (2001). Movement costs are the product of the allometric locomotion costs and twice the Euclidean distance to the core cell as the individual has to move to the foraging cell and back to the central place.

If the individuals daily energy requirements could already be satisfied, the home range formation is completed and the animal reduces the resources in all its home range cells according to its feeding rate and movement costs. Otherwise the addition of more cells to the home range continues.

In the last added cell the animal's resource gain may be higher than the resources it would need to meet its daily feeding rate. Should this be the case, the animal only consumes the amount needed to cover its requirements and leaves the remaining resources for later foragers.

While an individual is still searching for its home range and it can not meet its requirements even in the maximum possible home range it chooses a new core cell and starts a new home range search. During initialization individuals have 100 attempts to successfully establish a home range. Juveniles or dispersers have ten attempts to establish a new home range in their natal patch or new settlement patch respectively.

Established individuals maintain their chosen core cells and forage within their established home ranges. By simulating daily foraging behavior, we allow for the dynamic adjustment of home range sizes in response to factors like individual needs and competition-driven resource fluctuations.

The energy requirements of an individual are updated each day according to its reproductive state. This is necessary because the growing fetus as well as the suckling offspring exert substantial energy demands on the mother and the needed energy must largely be obtained from an increased food intake by pregnant or lactating females. During gestation the required energy increase compared to non-productive females is about 20 % and about 80 % during lactation (Oftedal (1985) in Gittleman and Thompson (1988)). This higher food intake for reproductive females is acknowledged in the model by adding the declared amount of food to their normal daily diet.

The daily foraging procedure broadly follows the home range search procedure. Individuals start foraging in their formerly chosen core cell and expand their foraging area around the center, reducing the resource level in each visited cell according to Equation (2). As soon as the resources provided by the added up cells can cover the animal's daily food intake, the individual stops feeding and all added cells are stored as its home range.

Individuals not able to find enough food in their maximum possible home range encounter an energy survival crisis. An individual's fasting endurance or survival time, which describes the potential to survive such a time of resource shortage, is positively correlated with body mass due to the consideration that the magnitude of stored fat becomes a greater fraction of body mass as size increases among mammals (Lindstedt and Boyce 1985). If an individual experiences consecutive days of food shortage and if this fasting period exceeds its allometric survival time after Lindstedt and Boyce (1985), the individual dies and is subsequently excluded from the simulation.

B) Reproduction. Reproduction is a deterministic model process following the principle of parsimony. All female individuals which reach maturity get pregnant based on the assumption that enough reproducing males are present. The age of maturity depends on the female's body size (Hamilton et al. 2011). Mature females get pregnant again if they are not lactating. Pregnant females that reach the end of their gestating period bear offspring. The size of an individual's litter is also an allometric trait (Hamilton et al. 2011). This process is reflecting the high production potential of many small mammals that are characterized by high rates of conception, early maturity, and short gestation periods with consecutive litters following in rapid succession (Golley et al. 1975). Offspring stay in the same burrow as the mother until the end of the lactation period. During this time they are not considered to have their own home range. The time of lactation scales allometrically with the mother's body mass after Hamilton et al. (2011). At independence, all juveniles inherit their mother's species and get their adult body mass from the species normal body mass distribution. All other traits are assigned as described in the section "Individuals and trait assignment". Juveniles that get old enough to

feed themselves (i.e. at the end of the lactation period) get ten attempts to successfully establish a home range in their natal patch. Unsuccessful juveniles will die, simulating juvenile mortality, and will not contribute to their mother's reproductive success.

C) Mortality. At the end of each day dead individuals are excluded from the simulation. Animals can face different causes of death:

1) Starvation mortality related to an insufficient food supply in the home range,
2) Age-related mortality
3) Natural mortality or
4) Density-dependent mortality

If individuals reach their maximum possible home range area and still can not cover their daily energy requirements they face a food shortage in their current home range. If consecutive days of resource shortage add up to a time period that exceeds an animal's fasting endurance, it dies due to an insufficient energy supply in its home range. If the period of energy shortage is still briefer than its fasting endurance, the individual does not die and has a new chance to satisfy its feeding needs on the next day, in its old home range. A successful day, when energy demands can be met within the home range, discontinues a fasting period, which is counted anew every time.

When an animal gets older as its maximum age it dies due to senility. The maximum age is a value drawn from a normal distribution around the individual's maximum lifespan with a standard deviation of 10% of the average.

Additionally to the above described causes of mortality, all individuals have a fixed, mass dependent daily mortality rate following a scaling law for natural mortality rates established by McCoy and Gillooly (2008). This probability of death is reflecting causes that are not explicitly modelled like diseases or predation.

To determine the probability of a density-related mortality the number of all individuals belonging to the same species is counted and compared with the patch capacity for this species which depends on the body mass. The probability of death due to negative density-dependent effects (e.g. intraspecific competition, predation, parasitism or disease transmission) increases when the number of conspecifics gets closer to the species' (allometric) maximum density (Equation (4)) and is calculated as follows:

$$p(D_c) < \frac{A_{Spec}}{C_{Spec}} \tag{3}$$

with: $p(D_c)$ = Probability of death due to high density
A_{Spec} = Abundance of species x
C_{Spec} = Capacity for species x

The patch capacity for each species depends on the body mass (M):

$$C_{Spec} = 450 \cdot M^{-0.75} \tag{4}$$

The dependence of population densities on mean adult body mass in herbivorous mammals was first evaluated by Damuth (1981). He found a scaling exponent of -0.75 for this relationship that was later supported (Peters and Wassenberg 1983). Silva and Downing (1995) could verify this allometric exponent for mammals with a body mass smaller than 100 kg. The intercept of the relationship between population density and body size was chosen after some model testing and is subject to a further model parameterisation.

Dispersal

After each patch completed the simulation of its population dynamics, the dispersal process within the metacommunity starts. In each patch a random subset of individuals is selected as dispersers each day. The actual number of daily dispersers depends on the overall dispersal propensity in the metacommunity, which can vary between 0 (philopatry) and 1 (mandatory dispersal). The dispersal propensity (pD_{life}) describes the probability of an individual to disperse during its lifetime. Daily dispersal probabilities (pD_{day}) are calculated for each species depending on their average lifetime in the simulations (e_0), which is a trait that strongly depends on the species' mean body mass. Daily dispersal probabilities in the model are calculated as follows:

$$pD_{day} = 1.0 - (1.0 - pD_{life})^{\frac{1}{e_0}} \tag{5}$$

with: pD_{day} = Daily dispersal probability
pD_{life} = Lifetime dispersal probability
e_0 = Species average life expectancy

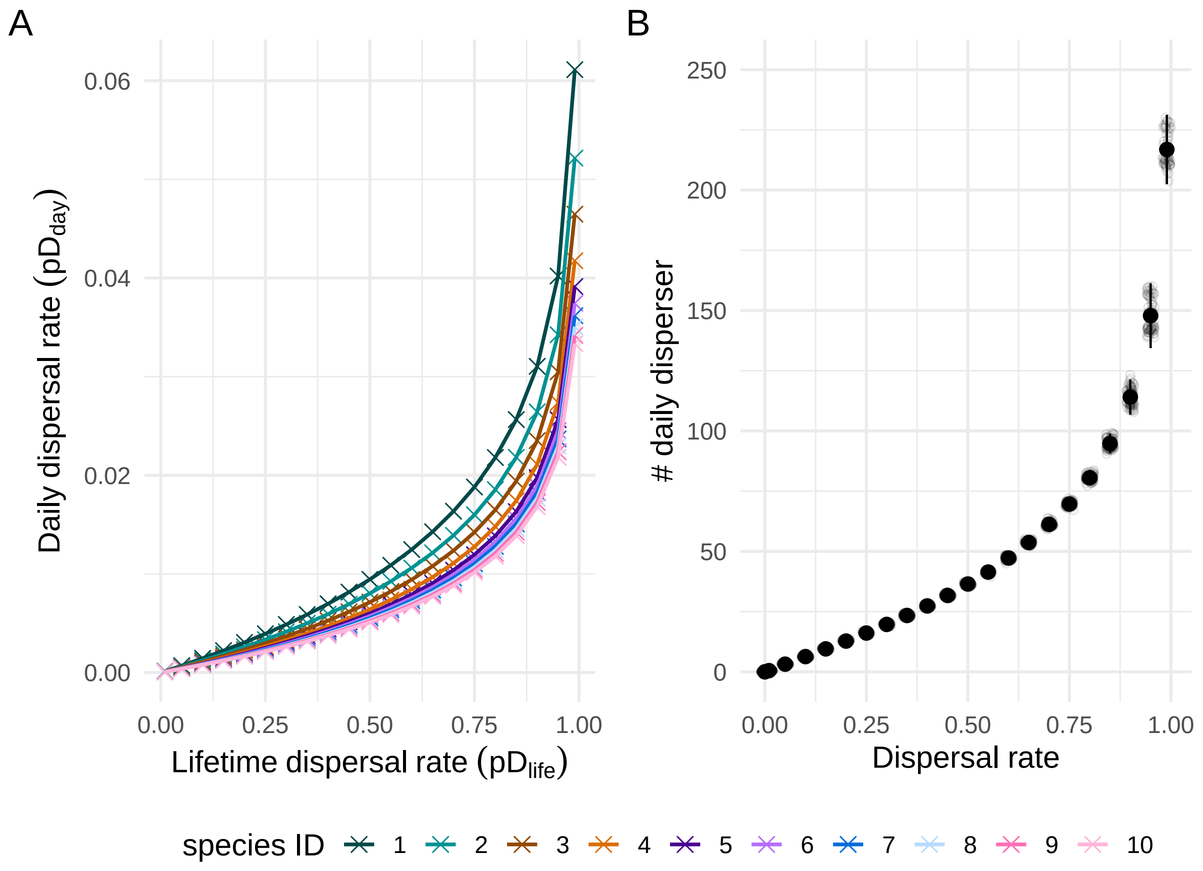

Figure S1.2: *(A) displays relationship between lifetime dispersal rate and daily dispersal rate for each species. (B) shows the resulting total number of daily dispersers for each dispersal propensity scenario.*

The model can distinguish between a context-independent emigration scenario and a context-dependent emigration scenario. A context-independent emigration simulates the same dispersal probabilities for individuals in all patches regardless of patch quality. In contrast, for the context-dependent emigration dispersal probabilities vary for resident individuals based on patch habitat quality, while maintaining a constant average daily number of dispersers, consistent with the simulated dispersal propensity. This means that individuals living in patches with currently poor habitat conditions show an increase in dispersal propensity, while individuals from currently high quality patches experience a lower dispersal probability.

Dispersers select a new patch to colonize based on their knowledge of habitat quality from a randomly chosen subset of the metacommunity. A random subset of patches is sampled from the metacommunity for each disperser, excluding the disperser's current patch. The number of patches within this subset is based on a disperser's prospecting effort, which can vary between 1 and M-1 and depends on the simulated prospecting effort scenario (see Table S1.3). This scenario determines how the prospecting effort will vary between individuals in the metacommunity and it can distinguish three separate cases:

A) Uniform prospecting effort. For a uniform prospecting effort, each individual in the metacommunity samples its environment (potential destination patches) with the same effort, i.e. n is equal for all dispersers. Prospecting effort n is varied for the whole metacommunity from 1, which resembles blind dispersal, to M-1 patches, which corresponds to complete knowledge of all metacommunity patches.

B) Interspecific differences in prospecting effort. A species-specific prospecting effort was implemented by correlating the trait with the mean body mass of a species, as it is associated with longer movement distances and a higher perceptual range (Kelt and Van Vuren 2001; Mech and Zollner 2002; Sutherland et al. 2000). Both allometrically scaled traits will increase a roaming animal's number of encountered, and therefore prospected, patches. Thus, in this scenario, larger species are considered to have a wider prospecting range than smaller species.

C) Intraspecific differences in prospecting effort. Even within the same species prospecting effort can vary substantially between individuals (Fielding et al. 2023). Intraspecific differences in prospecting effort are implemented by assigning each individual a random prospecting effort from a uniform distribution in the range of 1 to M-1. If prospecting effort is modelled as an inherited trait, offspring will inherit the prospecting effort from their mother displaying a certain variation around the parental trait. Otherwise offspring will also receive a random prospecting effort.

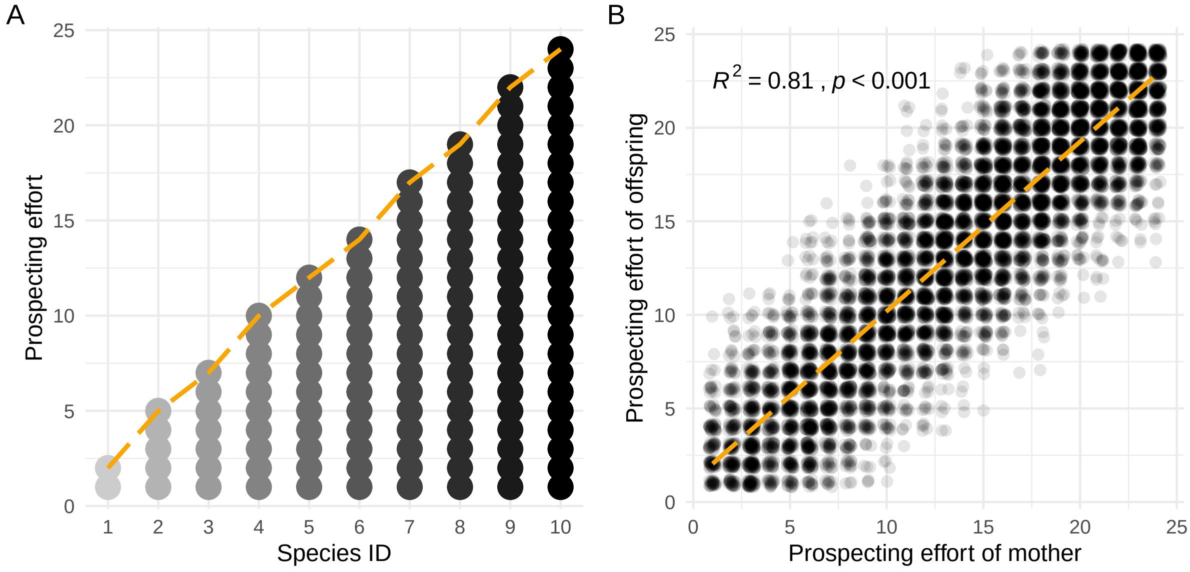

Figure S1.3: *(A) shows the species-specific prospecting effort for prospecting scenario B of interspecific differences. Prospecting effort is steadily increasing with the species average body mass. (B) shows the resulting correlation between mother and offspring trait in prospecting effort for prospecting scenario C of inherited intraspecific differences. Offspring prospecting effort varies around the maternal trait with sd = 3.*

The individual prospecting effort trait determines how many patches a disperser has sampled before dispersal, a higher prospecting effort leads to a larger subset of patches. Following the "best-of-n" strategy as described by Ponchon (2024) and Ponchon et al. (2021), dispersers will settle in the highest-quality patch within their individual subset.

Dispersers are assigned to the local community of their settlement patch and will establish a new home range based on the daily foraging routine (see subroutine *Foraging*) in the next time step. Dispersers unable to establish themselves in the new patch are counted as a failed dispersal to calculate overall dispersal success in the metacommunity.

Table S1.3: *Dispersal-related model parameters.*

Parameter	Level	Description
Number of patches (M)	25	Number of patches in the metacommunity
Extinction probability (pE)	0.002	Daily patch extinction probability
Dispersal probability (pD)	0, 0.1, 0.2, ..., 0.9, 0.99	Defines the probability of an individual to disperse
Prospecting scenario	Uniform, Interspecific, Intraspecific	Defines the differences in prospecting effort between individuals
Emigration scenario	Context-independent, Context-dependent	Emigration probability can either be independent of patch quality or vary between patches of different habitat quality
Movement mortality	None, 1 % per patch, 2 % per patch	Defines the mortality risk faced by dispersers per prospected patch
Cost of integration	Low, High	Defines the foraging order of dispersers and residents during initial establishment of dispersers

The dispersal procedure can include different costs for the dispersing individuals:

A) Cost of movement. Prospecting and dispersal movements both carry a risk of mortality for dispersing individuals, as they may expose them to predators or deplete their energy reserves (Bonte et al. 2012; Delgado et al. 2014). The cost of moving between patches in the metacommunity is simulated as a direct mortality risk for moving individuals. This risk is primarily influenced by the prospecting effort of the dispersers, with the probability of mortality increasing linearly with each patch visited during dispersal. Even in the absence of prospecting ($n = 1$) and, hence, random dispersal, there is a chance of death during dispersal. Three different levels of mortality were simulated:

1) No mortality
2) Low mortality (0.01 probability per patch)
3) High mortality (0.02 probability per patch)

B) Cost of integration. Dispersers face integration costs (Bonte et al. 2012) when settling into occupied, potentially crowded patches, demonstrating a high competition for resources. These costs are captured in the model by varying the foraging order between dispersers and residents during the initial establishment phase:

1) Random foraging order
2) Dispersers are last in the foraging order

Integration costs are increased by prioritizing resident foraging over disperser establishment. This reflects the competitive disadvantage of newcomers, facing increased competition and resource scarcity in densely populated areas. In contrast, a random foraging sequence, independent of dispersal status, simulates a less challenging integration scenario.

References

Bell, W. J. (1990). “Central place foraging”. In: *Searching behaviour: The behavioural ecology of finding resources.* Springer, pp. 171–187.

Bonte, D., H. Van Dyck, J. M. Bullock, A. Coulon, M. Delgado, M. Gibbs, V. Lehouck, E. Matthysen, K. Mustin, M. Saastamoinen, et al. (2012). “Costs of dispersal”. In: *Biological reviews* 87.2, pp. 290–312.

Buchmann, C. M., F. M. Schurr, R. Nathan, and F. Jeltsch (2011). “An allometric model of home range formation explains the structuring of animal communities exploiting heterogeneous resources”. In: *Oikos* 120.1, pp. 106–118.

— (2012). “Movement upscaled–the importance of individual foraging movement for community response to habitat loss”. In: *Ecography* 35.5, pp. 436–445.

Calder, W. A. (1996). *Size, function, and life history.* Courier Corporation.

Clobert, J., J.-F. Le Galliard, J. Cote, S. Meylan, and M. Massot (2009). “Informed dispersal, heterogeneity in animal dispersal syndromes and the dynamics of spatially structured populations”. In: *Ecology letters* 12.3, pp. 197–209.

Damuth, J. (1981). “Population density and body size in mammals”. In: *Nature* 290.5808, p. 699.

Delgado, M., K. Bartoń, D. Bonte, and J. Travis (2014). “Prospecting and dispersal: their eco-evolutionary dynamics and implications for population patterns”. In: *Proceedings of the Royal Society B: Biological Sciences* 281.1778, p. 20132851.

Fielding, A. H., D. Anderson, S. Benn, R. Reid, R. Tingay, E. D. Weston, and D. P. Whitfield (2023). “Substantial Variation in Prospecting Behaviour of Young Golden Eagles Aquila chrysaetos Defies Expectations from Potential Predictors”. In: *Diversity* 15.4, p. 506.

Gittleman, J. L. and S. D. Thompson (1988). “Energy allocation in mammalian reproduction”. In: *American zoologist* 28.3, pp. 863–875.

Golley, F. B., K. Petrusewicz, and L. Ryszowski (1975). *Small mammals: their productivity and population dynamics.* 5. Cambridge University Press.

Grimm, V., U. Berger, F. Bastiansen, S. Eliassen, V. Ginot, J. Giske, J. Goss-Custard, T. Grand, S. K. Heinz, G. Huse, et al. (2006). “A standard protocol for describing individual-based and agent-based models”. In: *Ecological modelling* 198.1-2, pp. 115–126.

Grimm, V., U. Berger, D. L. DeAngelis, J. G. Polhill, J. Giske, and S. F. Railsback (2010). “The ODD protocol: a review and first update”. In: *Ecological modelling* 221.23, pp. 2760–2768.

Grimm, V., S. F. Railsback, C. E. Vincenot, U. Berger, C. Gallagher, D. L. DeAngelis, B. Edmonds, J. Ge, J. Giske, J. Groeneveld, et al. (2020). “The ODD protocol for describing agent-based and other simulation models: A second update to improve clarity, replication, and structural realism”. In: *Journal of Artificial Societies and Social Simulation* 23.2.

Hamilton, M. J., A. D. Davidson, R. M. Sibly, and J. H. Brown (2011). “Universal scaling of production rates across mammalian lineages”. In: *Proceedings of the Royal Society of London B: Biological Sciences* 278.1705, pp. 560–566.

Haskell, J. P., M. E. Ritchie, and H. Olff (2002). “Fractal geometry predicts varying body size scaling relationships for mammal and bird home ranges”. In: *Nature* 418.6897, p. 527.

Jost, L. (2006). “Entropy and diversity”. In: *Oikos* 113.2, pp. 363–375.

Kelt, D. A. and D. H. Van Vuren (2001). “The ecology and macroecology of mammalian home range area”. In: *The American Naturalist* 157.6, pp. 637–645.

Lindstedt, S. L. and M. S. Boyce (1985). “Seasonality, fasting endurance, and body size in mammals”. In: *The American Naturalist* 125.6, pp. 873–878.

McCoy, M. W. and J. F. Gillooly (2008). “Predicting natural mortality rates of plants and animals”. In: *Ecology letters* 11.7, pp. 710–716.

Mech, S. G. and P. A. Zollner (2002). “Using body size to predict perceptual range”. In: *Oikos* 98.1, pp. 47–52.

Mitchell, M. S. and R. A. Powell (2004). “A mechanistic home range model for optimal use of spatially distributed resources”. In: *Ecological Modelling* 177.1-2, pp. 209–232.

— (2012). “Foraging optimally for home ranges”. In: *Journal of Mammalogy* 93.4, pp. 917–928.

Nagy, K. A. (2001). “Food requirements of wild animals: predictive equations for free-living mammals, reptiles, and birds”. In: *Nutrition Abstracts and Reviews, Series B.* Vol. 71. 10, 21R–31R.

Oftedal, O. (1985). “Pregnancy and lactation”. In: *Bioenergetics of wild herbivores.* CRC Press, pp. 215–238.

Orians, G. H. (1979). “On the theory of central place foraging”. In: *Analysis of ecological systems*, pp. 157–177.

Peters, R. H. and K. Wassenberg (1983). “The effect of body size on animal abundance”. In: *Oecologia* 60.1, pp. 89–96.

Ponchon, A. (2024). “Prospecting for informed dispersal: reappraisal of a widespread but overlooked ecological process”. In.

Ponchon, A., A. Scarpa, G. Bocedi, S. C. Palmer, and J. M. Travis (2021). “Prospecting and informed dispersal: Understanding and predicting their joint eco-evolutionary dynamics”. In: *Ecology and Evolution* 11.21, pp. 15289–15302.

Reed, J. M., T. Boulinier, E. Danchin, and L. W. Oring (1999). “Informed dispersal: prospecting by birds for breeding sites”. In: *Current ornithology*, pp. 189–259.

Rohwäder, M.-S., C. Gallagher, and F. Jeltsch (2024). “Variations in risk-taking behaviour mediate matrix mortality’s impact on biodiversity under fragmentation”. In: *Ecography*, e07140.

Rohwäder, M.-S. and F. Jeltsch (2022). “Foraging personalities modify effects of habitat fragmentation on biodiversity”. In: *Oikos* 2022.12, e09056.

Shingleton, A. (2010). “Allometry: the study of biological scaling”. In: *Nature Education Knowledge* 3.10, p. 2.

Silva, M. and J. A. Downing (1995). “The allometric scaling of density and body mass: a nonlinear relationship for terrestrial mammals”. In: *The American Naturalist* 145.5, pp. 704–727.

Sutherland, G. D., A. S. Harestad, K. Price, and K. P. Lertzman (2000). “Scaling of natal dispersal distances in terrestrial birds and mammals”. In: *Conservation ecology* 4.1.

Szangolies, L., M.-S. Rohwäder, and F. Jeltsch (2022). “Single large AND several small habitat patches: A community perspective on their importance for biodiversity”. In: *Basic and Applied Ecology* 65, pp. 16–27.

Teckentrup, L., V. Grimm, S. Kramer-Schadt, and F. Jeltsch (2018). “Community consequences of foraging under fear”. In: *Ecological Modelling* 383, pp. 80–90.

Whittaker, R. H. (1975). *Communities and ecosystems.* Macmillan.
