# Supplementary material for: How Prospecting for Informed Dispersal Shapes Biodiversity Patterns in a Metacommunity
Source: Ecol Evol. 2026 Jul 7;16(7):e73912. doi: 10.1002/ece3.73912 (PMC13341221; doi:10.1002/ece3.73912)
Supplement: Supplementary file 2 — Figure S2.1: Species reproductive success (a) and dispersal success (b) for different scenarios of prospecting effort and integration costs, showing the disproportionate impact on larger species, which have higher energy requirements and are particularly vulnerable to increasing competition. Figure S2.2: Gamma (γ) and alpha (α) species richness (a, b) and effective number of species (c, d), giving a more balanced impression of species composition. Due to the reducing effect of disturbed patches, α‐diversities (b, d) are consistently slightly lower than γ‐diversities (a, c). Otherwise, α‐ and γ‐diversities show the same patterns, as we simulated identical patches and no differences in habitat requirements between species. Variability in the effective number of species (e) between patches in the metacommunity is reduced as dispersal increases, due to the homogenising effect of dispersal. Informed colonisation and settlement further reduced variability due to rapid recolonisation of disturbed patches. Figure S2.3: Juvenile dispersal displays no negative effect of high prospecting effort on diversity due to the much lower number of daily dispersers. Thus, juvenile dispersers always experience less competition. Dispersal rate indicates the daily proportion of offspring that will disperse from their natal patch after reaching independence. Figure S2.4: We found no impact of a phenotype‐dependent dispersal on biodiversity in our metacommunity model. For a phenotype‐dependent dispersal, dispersers were either larger (dark blue) or smaller (light blue) than their respective species' average body mass compared to dispersers being of a random body mass (grey). [file ECE3-16-e73912-s001.pdf]

Appendix II

Additional results

1. Dispersal and reproductive success disaggregated by species

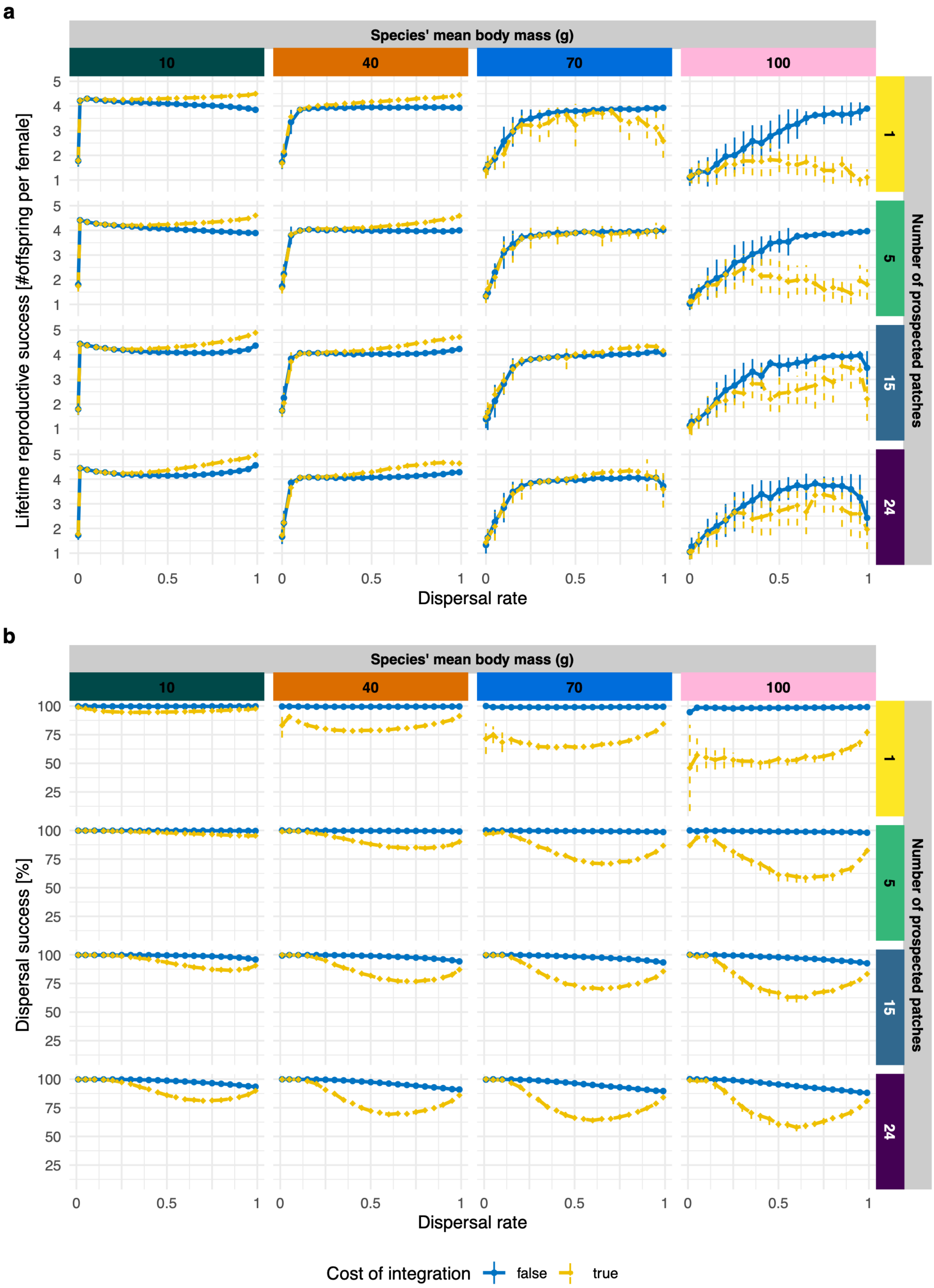

Figure S2.1 Species reproductive success (a) and dispersal success (b) for different scenarios of prospecting effort and integration costs, showing the disproportionate impact on larger species, which have higher energy requirements and are particularly vulnerable to increasing competition.

2. Different measures and scales of diversity

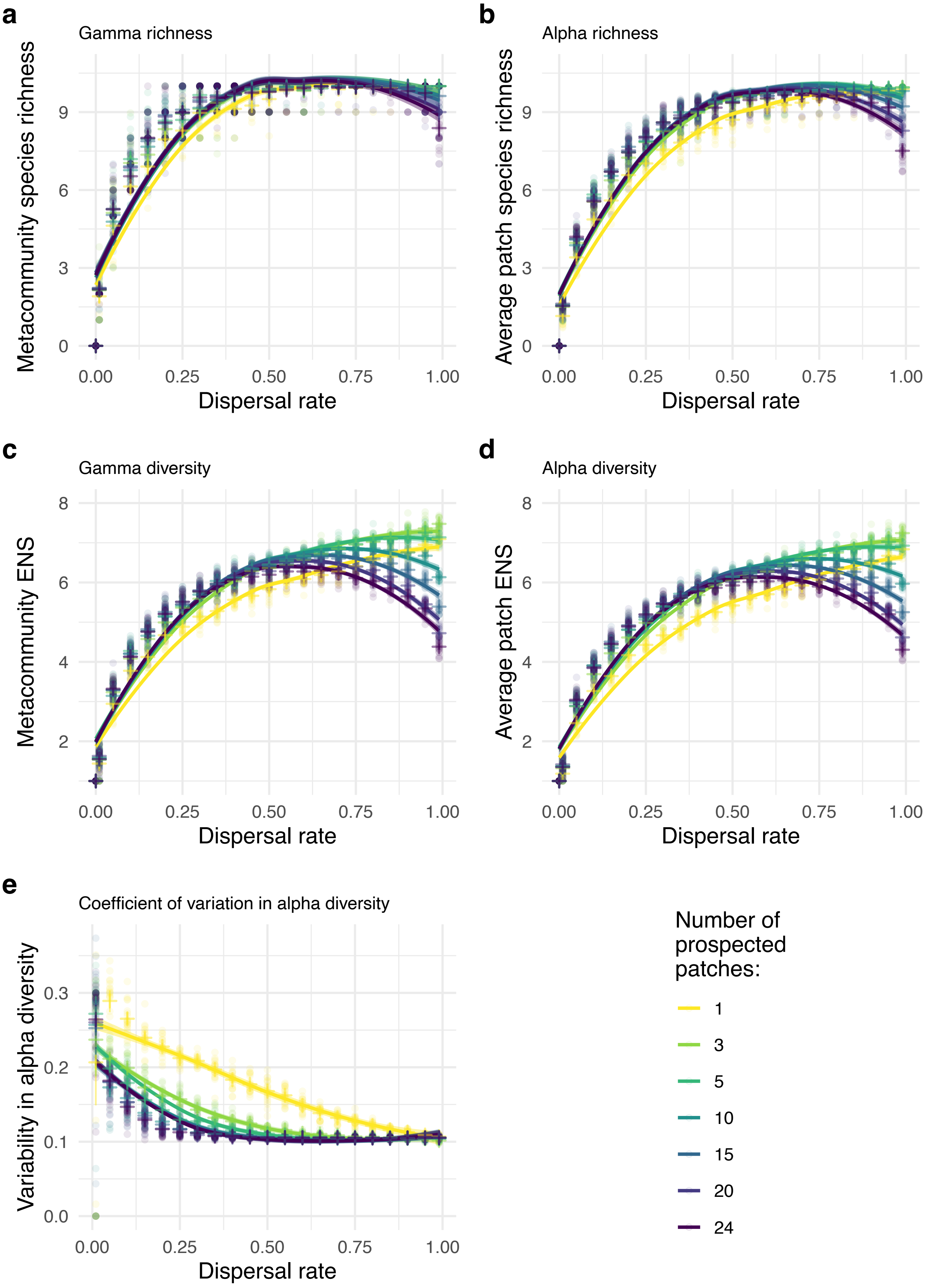

Figure S2.2 Gamma (γ) and alpha (α) species richness (a,b) and effective number of species (c,d), giving a more balanced impression of species composition. Due to the reducing effect of disturbed patches, α-diversities (b,d) are consistently slightly lower than γ-diversities (a,c). Otherwise, α- and γ-diversities show the same patterns, as we simulated identical patches and no differences in habitat requirements between species. Variability in the effective number of species (e) between patches in the metacommunity is reduced as dispersal increases, due to the homogenising effect of dispersal. Informed colonisation and settlement further reduced variability due to rapid recolonisation of disturbed patches.

Additional scenario

1. Juvenile dispersal

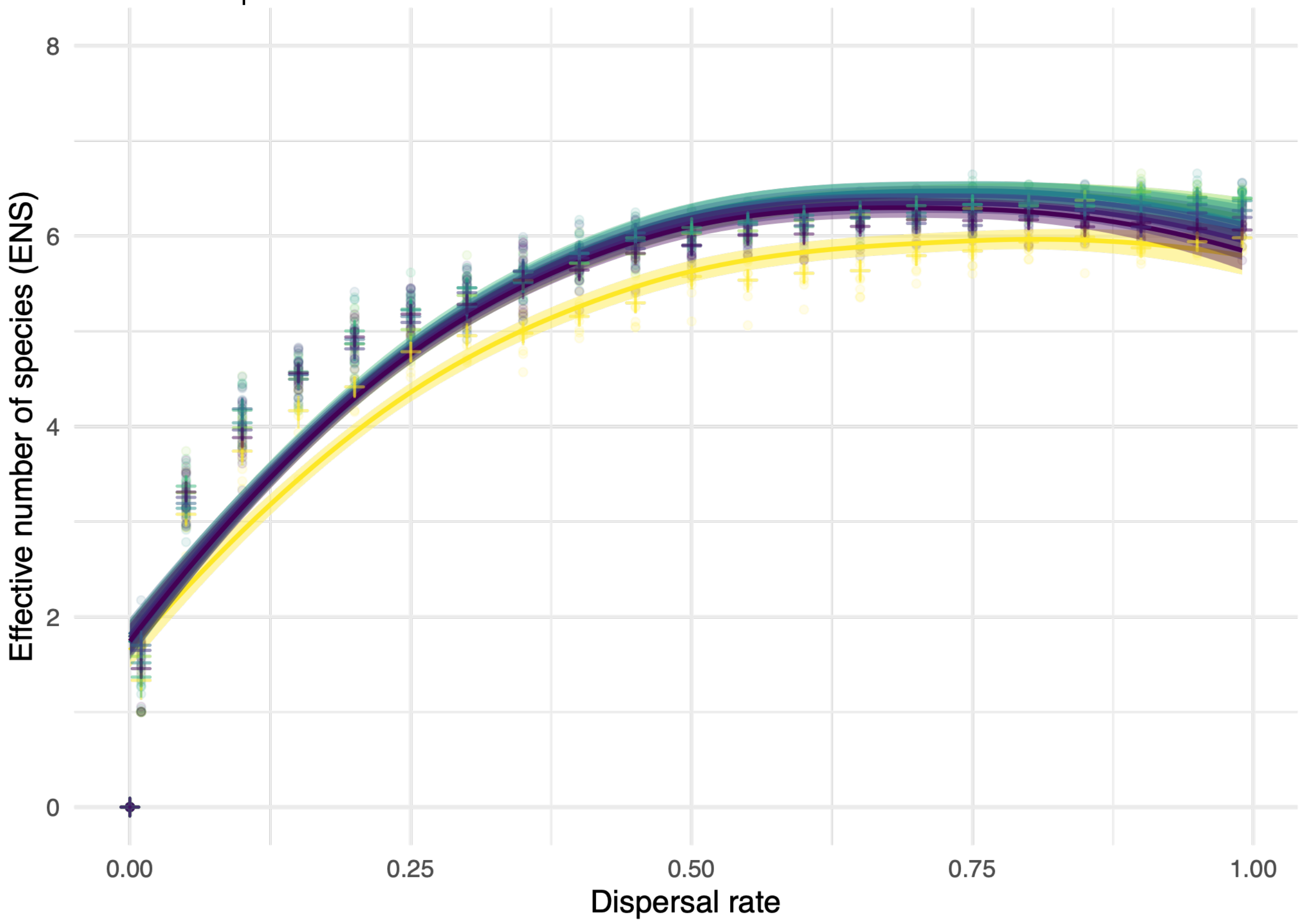

Figure S2.3 Juvenile dispersal displays no negative effect of high prospecting effort on diversity due to the much lower number of daily dispersers. Thus, juvenile dispersers always experience less competition. Dispersal rate indicates the daily proportion of offspring that will disperse from their natal patch after reaching independence.

2. Phenotype-dependent dispersal

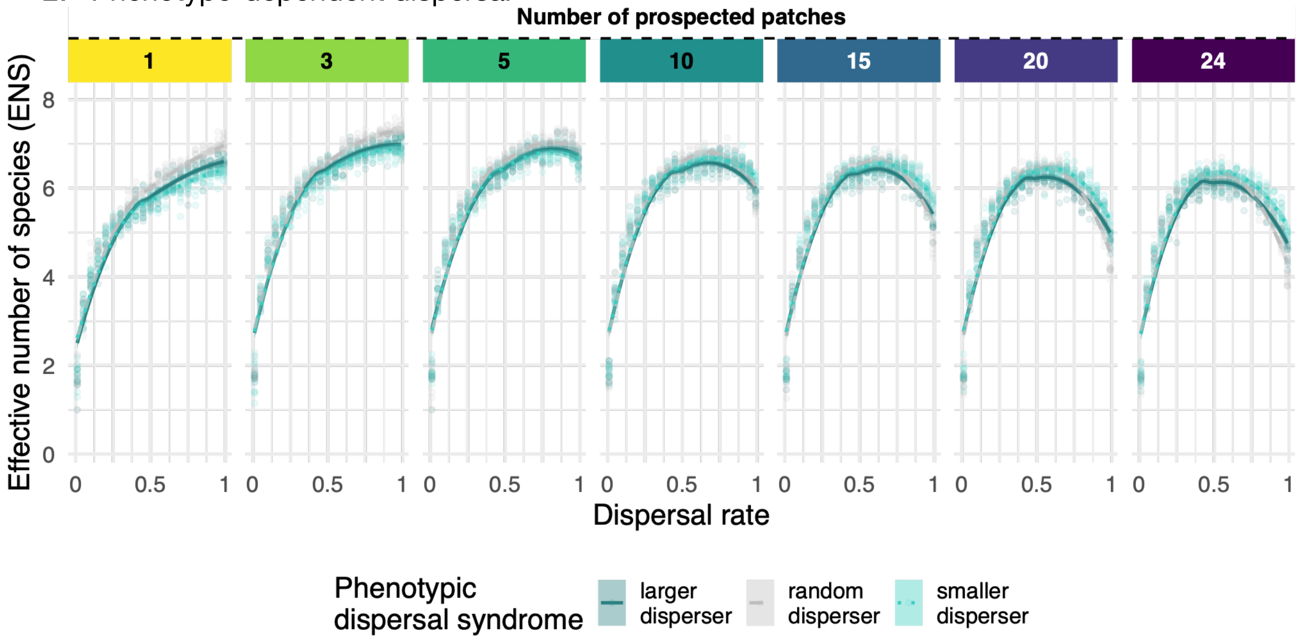

Figure S2.4 We found no impact of a phenotype-dependent dispersal on biodiversity in our metacommunity model. For a phenotype-dependent dispersal, dispersers were either larger (dark blue) or smaller (light blue) than their respective species' average body mass compared to dispersers being of a random body mass (grey).
